# Incorporating calendar effects to predict influenza seasonality in Milwaukee, Wisconsin

**DOI:** 10.1017/S0950268819001511

**Published:** 2019-09-11

**Authors:** Ryan B. Simpson, Tania M. Alarcon Falconi, Aishwarya Venkat, Kenneth H. H. Chui, Jose Navidad, Yuri N. Naumov, Jack Gorski, Sanjib Bhattacharyya, Elena N. Naumova

**Affiliations:** 1 Friedman School of Nutrition Science and Policy, Tufts University, Boston, MA, USA; 2 City of Milwaukee Health Department Laboratory (MHDL), Milwaukee, WI, USA; 3 Versiti Wisconsin, Blood Research Institute, Milwaukee, WI, USA

**Keywords:** Age, calendar effects, influenza, school holidays, surveillance

## Abstract

Social outings can trigger influenza transmission, especially in children and elderly. In contrast, school closures are associated with reduced influenza incidence in school-aged children. While influenza surveillance modelling studies typically account for holidays and mass gatherings, age-specific effects of school breaks, sporting events and commonly celebrated observances are not fully explored. We examined the impact of school holidays, social events and religious observances for six age groups (all ages, ⩽4, 5–24, 25–44, 45–64, ⩾65 years) on four influenza outcomes (tests, positives, influenza A and influenza B) as reported by the City of Milwaukee Health Department Laboratory, Milwaukee, Wisconsin from 2004 to 2009. We characterised holiday effects by analysing average weekly counts in negative binomial regression models controlling for weather and seasonal incidence fluctuations. We estimated age-specific annual peak timing and compared influenza outcomes before, during and after school breaks. During the 118 university holiday weeks, average weekly tests were lower than in 140 school term weeks (5.93 *vs.* 11.99 cases/week, *P* < 0.005). The dampening of tests during Winter Break was evident in all ages and in those 5–24 years (RR = 0.31; 95% CI 0.22–0.41 *vs.* RR = 0.14; 95% CI 0.09–0.22, respectively). A significant increase in tests was observed during Spring Break in 45–64 years old adults (RR = 2.12; 95% CI 1.14–3.96). Milwaukee Public Schools holiday breaks showed similar amplification and dampening effects. Overall, calendar effects depend on the proximity and alignment of an individual holiday to age-specific and influenza outcome-specific peak timing. Better quantification of individual holiday effects, tailored to specific age groups, should improve influenza prevention measures.

## Introduction

Despite global efforts to control influenza, this seasonal infection remains a serious health problem, especially in children, older and immuno-compromised peoples. Influenza viral infection is highly contagious and has a short incubation period yet is preventable if multiple barriers are employed. Increased social mixing, frequent physical contacts and high travel volumes are known to boost influenza transmission [1, 2, 3]. Social outings are shown to heighten the opportunities for influenza spread, especially in susceptible populations such as children and the elderly [4, 5]. In contrast, school closures are associated with reductions of influenza incidence in school-aged children [6, 7]. The effects of holidays and social events on seasonal influenza have been explored in influenza surveillance time series and agent-based modelling studies [2, 7–10]. While these studies often account for holidays and mass gatherings, the differential and age-specific effects of school breaks, sporting events and national and cultural observances are not fully explored. Particularly, it is unclear whether such effects are uniform across age groups.

Most research is emphasizing understanding transmission dynamics, both in the context of socially acceptable control measures such as school holiday timing or duration and quantifying transmission related to travel timing, volume and route [6, 7, 11–16]. A spatiotemporal study of influenza spread among school-aged children in Belgium illustrated that changes in mixing patterns, rather than changes in travel behaviour, are responsible for altering the seasonal pattern of influenza [7]. Another study showed that during school closures, influenza risk shifts from school-aged children to adults due to increased child–adult interactions [3], supporting that routine vaccination of children might impart indirect protection to the elderly [17]. Stochastic simulation studies have suggested that weekends and holidays could delay seasonal disease peaks and mitigate infection spread by periodically dampening transmission [7, 12]; for example, an extension of the Christmas holiday by 1 week might further mitigate infection [7, 12]. While Christmas holidays have the largest documented impact on influenza transmission, other school breaks may also reduce an epidemic's size, stressing the importance of full calendar analyses [3, 11–13].

Contrary to school closures and other events likely to reduce social mixing, large sporting events, festivities and mass gatherings facilitate the spread of infections [4, 5, 18, 19]. In Singapore, the Asian Youth Games of 2009 caused the declaration of a public health emergency due to the spread of A(H1N1) influenza among adolescents [20]. In fact, Singaporean health officials implemented strict control measures such as airport screening, quarantining hospitalised H1N1-infected persons and their close contacts, and mandatory vaccination of all youth competitors [20]. Mass gatherings involve increased travel of diverse groups from a wide range of ages and countries-of-residence and can promote an introduction of novel viruses to local communities. These novel viruses can cause substantial, complex and unpredictable effects on influenza activity and escape traditional surveillance detection tools. Authorities may underestimate the true influenza incidence at these gatherings, as shown during the 2008 World Youth Day in Australia [21] and the American Super Bowl from 1974 to 2009 [4]. Using county-level vital statistics of the USA from 1974 to 2009, researchers demonstrated that social mixing during the Super Bowl affects influenza dissemination in population groups. Authors estimated having a local team in the Super Bowl caused an 18% increase in influenza deaths for those over age 65, with no impacts on influenza mortality in hosting cities. The effect was most pronounced in years when the dominant influenza strain was more virulent or when the Super Bowl occurred closer to the peak of the influenza season [4].

Holiday-related travel also increases social contacts between infected and susceptible persons of all ages. Although the travel patterns themselves may not directly influence transmission, increased travel volumes "affect the coupling force between epidemics in different (sub-populations) and the opportunity for individuals to be exposed to the disease" [7]. Due to winter seasonal migration among older adults from colder to warmer states, in several southern states, such as California, Arizona, Texas and Florida, the proportion of non-residents being hospitalised for pneumonia and influenza (P&I) was higher in winter months than summer months, although for most states, the opposite was true. In Florida, the proportion of all P&I hospitalisations attributable to out-of-state residents was over three times as high between October and March compared to the usual nadir of influenza activity from April to September. A large portion of out-of-state resident P&I hospitalisations in Florida are derived from Northeastern and Midwestern states, such as New York, Michigan, Pennsylvania and Ohio [8].

In-depth analyses assessing travel-related transmission and the effects of holidays and social events on seasonal influenza have been explored largely in agent-based modelling studies [3, 7, 12]. A simulation study showed that higher travel volumes could shift the peak timing of influenza epidemics earlier compared to national holiday observances and school closures [3]. Shi *et al*. also noted that the impact of a public gathering on influenza prevalence depends on time proximity to the epidemic peak. Specifically, mass gatherings that occur within 10 days before the epidemic peak can result in as high as a 10% relative increase in the peak prevalence and the total attack rate and may have even worse impacts on local communities and travellers' families [2]. Furthermore, if 25% of the population travelled 1 day prior to a mass gathering that aligned with influenza peak incidence for that year, the prevalence of positives would increase approximately 11% (equivalent to an additional 63502 individuals infected), disproportionately targeting children and the elderly [2].

While simulation studies are exploring how social mixing affects influenza spread, time series analysis allows researchers to track the seasonal peaks of influenza outcomes [1, 9, 10]. On a national scale, influenza peak timing migrates both spatially [22] and temporally [9]. For example, for the influenza seasons 1991–2004, the hospitalisation rates for P&I in US older adults peaked in Western states (such as Nevada, Utah and California) earlier than in New England states (such as Rhode Island, Maine and New Hampshire). On a regional scale, the difference in peak timing is noticeable for different influenza strains [9]. In Wisconsin specifically, Lofgren *et al*. showed that influenza mortality peak timing occurred earlier in children and infants (calendar week 27) than the elderly (calendar week 30) from 1967 to 2004. That said, mortality rates were 10 times greater among the elderly than children and infants with differing rates across influenza A strains [1].

While the age-specific differences in influenza incidence are well documented, the potential contribution of regular calendar events, such as school breaks, sporting events and national and cultural observances to these differences, is largely unknown. Prior research suggests that such effects could vary across age groups, influenced by a holiday type, and impacted by proximity and alignment of influenza peak timing in relation to the holiday. As such, our research aimed to evaluate and quantify the effects of calendar events (in this case, holidays) on influenza seasonal signatures. In this study, we examined the impact of school holidays, religious observances, federal observances and sporting events for six age groups (all ages, ⩽4, 5–24, 25–44, 45–64, ⩾65 years) on four influenza health outcomes (tests, positives, influenza A and influenza B) in Milwaukee, Wisconsin, in 2004–2009 during routine laboratory surveillance. First, we compared averages of weekly outcomes as they occurred during holiday and non-holiday time periods. Next, we estimated the direction and magnitude of calendar effects using negative binomial regression models adjusted for meteorological conditions and seasonality. We then estimated annual peak timing for each outcome and evaluated differential effects of school holidays for five adjacent time periods: before and during Winter Break, between Winter and Spring Breaks, and during and after Spring Break. Finally, we produced age-specific seasonal signature curves to illustrate the dampening and amplification of tests during Winter and Spring Breaks. Through these time series-based methods, we explored possible age- and outcome-specific holiday effects and offer recommendations for influenza near-term forecast modelling.

## Methods

### Study area and health outcomes

**Fig. 1.**
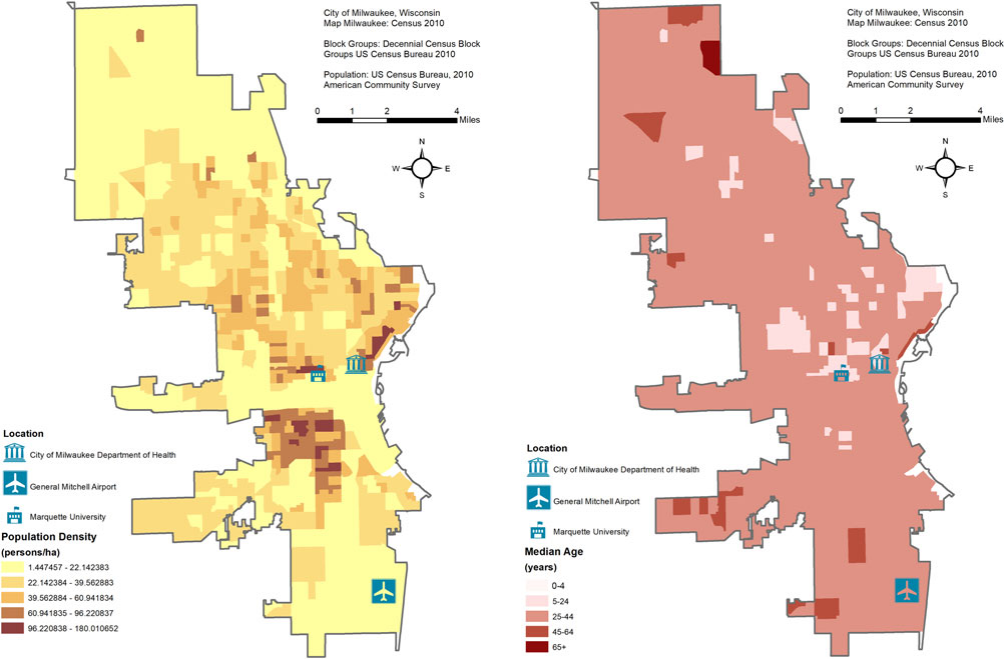
Maps of the study area's population density and average age with the location of the City of Milwaukee Health Department Laboratory (MHDL), General Mitchell International Airport and Marquette University.

Maps of the study area's population density and average age are shown in Figure 1. The City of Milwaukee Health Department Laboratory (MHDL), Wisconsin routinely collects tests from the students' residents of metropolitan areas and vicinities around Marquette University (MU). If confirmed positive for influenza, the MHDL classifies cases according to the Centers for Disease Control and Prevention (CDC) guidelines for subtypes: H1N1, H3N2, A (unknown subtype), B (Victoria and Yamagata subtypes), A (2009 H1N1) and variant of influenza A of swine origin (H3N2v). We obtained weekly counts of tests from the MHDL from 16 May 2004 to 19 December 2010 (344 weeks). We excluded the last 86 weeks (26 April 2009 to 19 December 2010) due to irregular surges of testing and positives during the 2009 Milwaukee epidemic due to the novel influenza A(H1N1) virus of swine origin. For the remaining 258 weeks (16 May 2004 to 25 April 2009), we considered four outcomes in our dataset: number of tests, tests that were confirmed positive, tests that were positive for influenza A and tests that were positive for influenza B. All recorded weekly counts for each of the four outcomes were grouped into six age categories: all ages, ⩽4, 5–25, 25–44, 45–64 and ⩾65. We combined counts for the <1 and 1–4 age groups into a ⩽4 category due to low numbers and we excluded four tests due to unknown age. The final dataset contained records aggregated into four outcomes (tests, positives, influenza A and influenza B), and six age categories with no missing data.

### Meteorological data

Meteorological data are routinely collected by a monitoring station at the General Mitchell International airport (KMKE/MKE) located ~7.5 miles from the MHDL (Fig. 1). We downloaded daily records of temperature, humidity and dewpoint from an open source website [23] and aggregated them into weekly averages. Sunday was designated as the beginning of each week to align with MHDL influenza data. Dewpoint values, which represent the perceived ambient temperature corrected for the air moisture content, were used in regression models.

### Calendar of selected events

For this study, we focus on major school holidays, religious observances for three major denominations (Christian, Jewish and Muslim), national federal observances and major sporting events. Holidays included were not exhaustive and span multiple calendar years. Durations of the holidays considered are shown in Supplementary Table S1. Holidays with fixed dates occur on the same day (e.g. New Years on January 1) or week (e.g. President's Day on 8th calendar week) annually. In contrast, floating holidays occur annually on different days or weeks of the year, and include the Jewish and Muslim religious observances, which do not follow the Gregorian calendar. We classified a holiday week as having at least 1 day of a holiday occurring during that week.

Information on school holidays was obtained from publicly available archives of semester-long academic calendars for MU and annual academic calendars for the Milwaukee Public School District [24, 25]. Undergraduate full-time student calendars with four consistently non-overlapping school holidays (i.e. Winter, Spring, Summer and Autumn Breaks) were closely aligned with the Milwaukee Public School district (see Supplementary Table S1). The exploratory analysis was performed using calendars from both sources. University-based calendars were chosen to represent school holidays for the entire 5–24 years age group for the final analysis.

Major annually occurring religious observances listed on the open source website, Time and Date [26], were selected based on religious preference in Milwaukee retrieved from the Association of Religion Data Archives [27]. All individual Christian and Jewish holidays were non-overlapping, while individual Muslim holidays had some co-occurrences annually.

Federal observances included nine individual holidays with fixed and floating dates retrieved from the Time and Date public database [26]. We selected five major sporting events: the Super Bowl (American football), the Triple Crown (horse races), the World Series (baseball), the NBA Finals (basketball) and the AHL Finals (hockey) to account for the professional sports teams (Green Bay Packers, Brewers, Bucks and Admirals for the national football, baseball, basketball and hockey leagues, respectively) based in Milwaukee, Green Bay and Wisconsin as well as major US national sporting events. Dates of individual sporting events were retrieved from two publicly available websites [26, 28]. Each of the five individual sporting events varied in duration annually depending upon the sport.

### Exploratory statistical analysis

A calendar week was coded 1 if a holiday took place during that week and 0 otherwise. First, we compared averages of weekly counts for each age group and outcome as they occurred during holiday and non-holiday time periods for each holiday category (school holidays, observances for three major religions, federal observances and sporting events) and for each holiday within a category (see Supplementary Table S1). We estimated the summary statistics for each health outcome and each age group (see Supplementary Table S2), used a non-parametric Mann–Whitney (MW) *U* test (significant differences defined *a priori* as *α* ⩽ 0.05) (see Supplementary Table S3), and determined the events for which we had sufficient counts of tests and positives. This allowed us to reduce the number of holidays for in-depth analyses.

### Direction and magnitude of the calendar effects

Next, we estimated the direction and magnitude of the calendar effects using negative binomial regression models adjusted for meteorological conditions and seasonality. We constructed the models sequentially to observe the contribution of individual factors, e.g. holidays, meteorological conditions and linear and seasonal trend components. Models were as follows:

Model 1: ln[*E*(*Y*_*tj*_)] = *β*_0_ + *β*_1_*H*_*t*_

Model 2: ln[*E*(*Y*_*tj*_)] = *β*_0_ + *β*_1_*H*_*t*_  + *β*_2_*D*_*t*_

Model 3: ln[*E*(*Y*_*tj*_)] = *β*_0_ + *β*_1_*H*_*t*_ + *β*_2_*D*_*t*_ + *β*_3_*t* + *β*_4_(sin(2*πωt*))

+ *β*_5_(cos(2*πωt*))

where *Y*_*tj*_ – values of the study outcome for *t*-week and *j*-age group; *H*_*t*_ – a binary time series indicating flagged weeks referring to either the six holiday categories (School, Christian, Jewish, Muslim, Federal, Sporting) or individual non-overlapping holidays selected for in-depth analysis; *D*_*t*_ – weekly dewpoint averages; *t* – refers to a time series indicating the consecutive study week from 1 to 258. Two periodic terms define seasonal oscillations with a frequency of *ω* = 1/*M*, where *M* = 52.25 represents the length of the annual cycle in weeks. The estimates of *β*_1_ regression coefficients and their error values were used to calculate the relative risk (RR) estimates along with their confidence intervals (95% CI): RR = exp{*β*_1_} and 95%CI RR 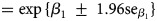. A dampening effect was defined as significant RR<1, while amplification effects were defined as significant RR > 1.

### Peak timing estimation

We estimated peak timing using Model 4, as follows:

Model 4: ln[*E*(*Y*_*tj*_)] = *β*_0_ + *β*_*t*_*t* + *β*_*s*_(sin(2*πωt*)) + *β*_*c*_(cos(2*πωt*))

We applied Model 4 to all tests, all positives, influenza A and influenza B for each age group over the 258-week study period. We estimated peak timing and its variance using equations provided in Table 1. Equations were derived by MacNeill and Naumova (2006) with further modifications by Alarcon-Falconi (2017) and were used to calculate age- and influenza outcome-specific peak timing estimates [29, 30]. To investigate how the school holidays modified the estimated peak timing, we extended Model 4 by adding four indicator variables – one for each holiday (Model 5). To adjust the estimates for meteorological conditions, we used Model 3.

**Table 1.** Peak timing equations for log-linear negative binomial regression models

Feature	Log-linear model
Regression model	ln(*E* [*Y*_*t*_]) = *β*_0_ + *β*_1_(t) + *β*_*s*_(sin(2*πωt*)) + *β*_*c*_(cos(2*πωt*))
Shift 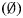	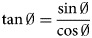 , thus: 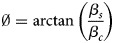 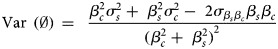
Peak timing (*P*_T_)	if 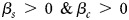 , then: 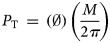 if *β*_*c*_ < 0, then: 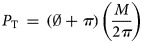 if 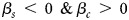 , then: 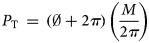 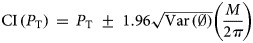

### Seasonal signatures

Finally, we explored seasonal signatures of influenza incidence with respect to the Winter and Spring university-based school breaks. Specifically, we compared five periods: period 1 – before Winter Break (5 weeks), period 2 – during Winter Break (4–6 weeks), period 3 – between Winter and Spring Breaks (6–9 weeks), period 4 – during Spring Break (2–3 weeks) and period 5 – after Spring Break (5 weeks). For each period, we calculated weekly average counts with pooled standard errors. The differences in average counts between adjacent time periods were tested with a paired *t* test. To derive the signature curves, we plotted the predicted values from Models 3, 4 and 5 for the interval between week 43 and week 23 of the following year. To demonstrate the effect of Winter and Spring Breaks, we interpolated the predicted values by connecting the first and last weeks of the holiday.

All statistical analyses were conducted using STATA (SE 15.1) software.

## Results

Over 258 study weeks, 2378 tests were submitted with 505 (21%) laboratory-confirmed positives including 410 (81%) influenza A (73 (18%) not subtyped) and 95 (19%) influenza B (see Supplementary Table S2). Cases were not distributed evenly across age groups. The largest portion of tests (1153 or 48%) belonged to the 5–24 years age group, which also had the most total (326 or 65%), influenza A (272 or 66%) and influenza B (54 or 57%) positives. The youngest age group (⩽4 years) had the fewest reported positives (12 or 2%) while the oldest age group had the fewest reported tests (178 or 7.5%). High values of skewness and kurtosis are indicative of seasonal spikes of weekly counts and a low number of cases; thus, the detailed analysis is limited to tests and total positives to ensure sufficient records.

**Fig. 2.**
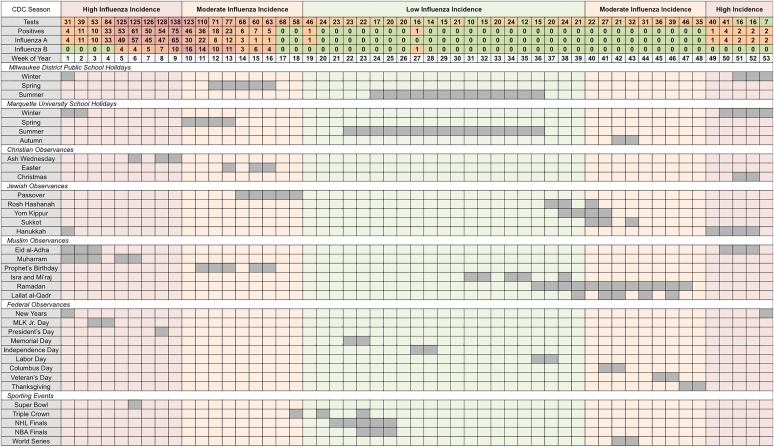
Total weekly counts for influenza outcomes (according to seasonal intensities) and an overview of school holidays', national and religious observances' and sporting events' typical weeks of occurrence (in chronological order for each holiday category) in Milwaukee, WI (2004–2009).

Next, we summed weekly cases by calendar week and aligned them with the selected holidays as they typically occur. The distribution of aggregated cases per calendar week and the overlap across holiday groups is shown in Figure 2, where we listed each individual holiday and when it approximately fell across 53 weeks of the year. The total counts of tests, positives, influenza A and influenza B were misaligned with periods of low-, moderate- and high-influenza incidence according to the CDC's nationally defined influenza season [31]. CDC-defined periods of high incidence occur in the first 9 and last 5 calendar weeks, while moderate incidence ranges over the 10th–18th and 40th–48th calendar weeks. The peak in distributions of observed cases has shifted towards the period of moderate incidence.

University and public-school holidays are well distributed throughout the year with Winter Break occurring during high-incidence periods, Spring and Autumn Breaks occurring during moderate incidence periods, and Summer Break occurring during low-incidence periods. Three national observances (New Years, Martin Luther King Jr. Day and President's Day), five religious observances (Ash Wednesday, Christmas, Hanukkah, Eid al-Adha and Muharram) and one sporting event (Super Bowl) occur during the high-incidence period (see Supplementary Table S3). During the CDC-defined low-incidence period, only 422 tests were submitted with two reported positives, one each for influenza A and B. During the moderate-incidence period, 987 tests were submitted and 141 (14%) were positive, all occurring during overlapping weeks of Spring Break, Easter, Passover and the Prophet's Birthday. During the high-incidence period, 969 tests were submitted including 362 (37%) positives, nearly all of which (92%) were influenza A. Weeks associated with Spring Break and the Prophet's Birthday had 64% of all influenza B positives observed across the study period. Individual holidays with at least three positive tests were selected for in-depth analysis (see Table S3).

### Direction and magnitude of the calendar effects

**Fig. 3.**
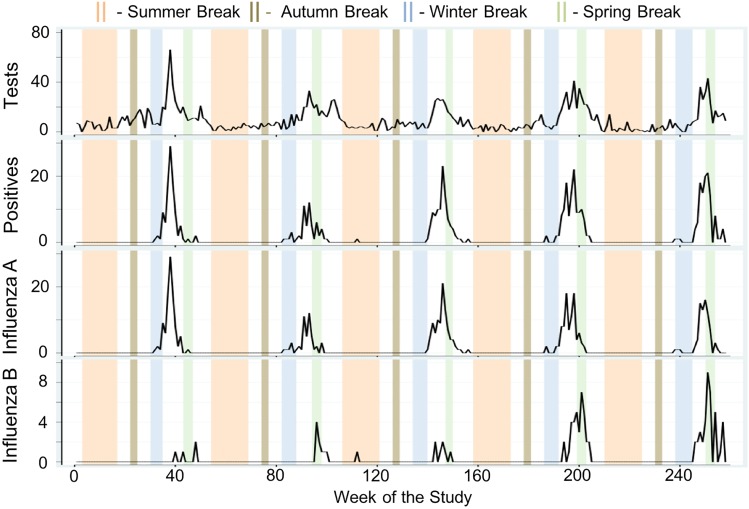
Time series of total weekly counts for four influenza outcomes (tests, positives, influenza A and influenza B) for all ages with superimposed university school holiday occurrences in Milwaukee, WI (2004–2009).

Next, we formally compared average weekly cases across holiday categories using MW *U* tests and examined the direction and magnitude of the calendar effects using negative binomial regression models adjusted for meteorological conditions and seasonality. Weekly tests, positives, influenza A and influenza B are shown in Figure 3 as time series plots with superimposed university-based school holiday occurrences. During the 118-week university-based school holiday period, the average number of tests was two times lower than during the 140-week school term (non-holiday) period (5.93 ± 6.68 *vs.* 11.99 ± 10.18 cases/week, *P* < 0.005). Similarly, during the 90-week public-school holiday period, the average number of tests was 60% lower than during the 168-week school term period (4.71 ± 4.70 *vs.* 11.63 ± 10.15 cases/week, *P* < 0.005). Similarly, average weekly positives were lower for both the university-based and public-school holiday compared to school term periods (0.85 ± 2.82 *vs.* 2.89 ± 5.66 cases/week, *P* < 0.005 and 0.42 ± 1.41 *vs.* 2.78 ± 5.55 cases/week, *P* < 0.005, respectively). Tests and positives reach near zero counts during the Summer and Autumn Breaks owing to the natural seasonal nadir of influenza during these times. The same patterns were observed for influenza A and B.

The results of direct comparison based on the MW tests for school holidays are shown in Table 2. In all instances when the MW test indicated a significant difference in average counts between the school holiday and school term periods, there is a dampening effect expressed as a negative percent change of average weekly cases. Age-specific holiday effects are prominent among school children (5–24 years), young adults (25–44 years) and the elderly (⩾65 years). While the magnitude of these dampening effects is somewhat greater when using the public-school calendar compared to the university school calendar for all age groups, the difference is marginal. In the 5–24 years age group, there is a 67% decrease in weekly tests (2.14 ± 3.63 *vs.* 6.44 ± 7.04, *P* < 0.005), a 74% decrease in influenza positives (0.49 ± 2.22 *vs.* 1.91 ± 4.02, *P* < 0.005), an 82% decrease in influenza A (0.31 ± 1.25 *vs.* 1.68 ± 3.71, *P* < 0.005) and a 25% decrease in influenza B (0.18 ± 1.06 *vs.* 0.24 ± 0.62, *P* = 0.01) using the university-based calendar. By applying the public-school calendar to the same age groups, we observed similar effects: 75%, 94% and 96% decrease in weekly tests, influenza positives and influenza A, respectively, and a notable decline of 79% for influenza B.

**Table 2.** Weekly counts, averages and percent change for the study, school term and school holiday periods for the university and public-school calendars for influenza tests, positives, influenza A and influenza B across six age groups in Milwaukee, WI in 2004–2009

	All study period			University academic calendar								Public-school district academic calendar							
				School term			School holiday			MW test	percent change^a^	School term (168 weeks)			School holiday (90 weeks)			MW test	Percent change^a^
	(258 Weeks)			(140 Weeks)			(118 Weeks)												
	Counts	Mean	s.d.	Counts	Mean	s.d.	Counts	Mean	s.d.	*P*-value	%	Counts	Mean	s.d.	Counts	Mean	s.d.	*P*-value	%
Tests																			
All ages	2378	9.22	9.24	1678	11.99	10.18	700	5.93	6.68	<0.005	−51	1954	11.63	10.15	424	4.71	4.70	<0.005	−60
⩽4	527	2.04	2.07	316	2.26	2.16	211	1.79	1.94	0.08	−21	380	2.26	2.11	147	1.63	1.93	0.08	−28
5–24	1153	4.47	6.12	901	6.44	7.04	252	2.14	3.63	<0.005	−67	1019	6.07	6.94	134	1.49	2.01	<0.005	−75
25–44	291	1.13	1.70	208	1.49	1.94	83	0.70	1.24	<0.005	−53	241	1.43	1.85	50	0.56	1.18	<0.005	−61
45–64	227	0.88	1.66	133	0.95	1.39	94	0.80	1.95	0.04	−16	174	1.04	1.64	53	0.59	1.68	0.04	−43
⩾65	178	0.69	1.74	120	0.86	2.05	58	0.49	1.27	0.02	−43	139	0.83	1.96	39	0.43	1.20	0.02	−93
Positive																			
All ages	505	1.96	4.68	405	2.89	5.66	100	0.85	2.82	<0.005	−71	467	2.78	5.55	38	0.42	1.41	<0.005	−85
⩽4	12	0.05	0.23	11	0.08	0.30	1	0.01	0.09	0.01	−88	12	0.07	0.28	0	0.00	0.00	0.01	−100
5–24	326	1.26	3.39	268	1.91	4.02	58	0.49	2.22	<0.005	−74	315	1.88	4.07	11	0.12	0.42	<0.005	−94
25–44	87	0.34	0.92	66	0.47	1.10	21	0.18	0.62	0.01	−62	76	0.45	1.08	11	0.12	0.42	0.01	−73
45–64	32	0.12	0.56	20	0.14	0.56	12	0.10	0.56	0.20	−29	22	0.13	0.52	10	0.11	0.63	0.20	−15
⩾65	47	0.18	1.03	40	0.29	1.37	7	0.06	0.27	0.09	−79	41	0.24	1.26	6	0.07	0.29	0.09	−71
Influenza A																			
All ages	410	1.59	4.10	349	2.49	5.20	61	0.52	1.63	<0.005	−79	391	2.33	4.91	19	0.21	0.61	<0.005	−91
⩽4	11	0.04	0.22	10	0.07	0.28	1	0.01	0.09	0.02	−86	11	0.07	0.27	0	0.00	0.00	0.02	−100
5–24	272	1.05	2.93	235	1.68	3.71	37	0.31	1.25	<0.005	−82	266	1.58	3.52	6	0.07	0.25	<0.005	−96
25–44	67	0.26	0.81	54	0.39	0.99	13	0.11	0.47	0.01	−72	59	0.35	0.96	8	0.09	0.32	0.01	−74
45–64	21	0.08	0.38	16	0.11	0.48	5	0.04	0.20	0.30	−64	18	0.11	0.45	3	0.03	0.18	0.30	−73
⩾65	38	0.15	0.97	34	0.24	1.30	4	0.03	0.18	0.08	−88	36	0.21	1.19	2	0.02	0.15	0.08	−90
Influenza B																			
All ages	95	0.37	1.20	56	0.40	1.02	39	0.33	1.38	0.02	−18	76	0.45	1.28	19	0.21	1.00	0.02	−53
⩽4	1	0.00	0.06	1	0.01	0.08	0	0.00	0.00	0.36	−100	1	0.01	0.08	0	0.00	0.00	0.36	−100
5–24	54	0.21	0.85	33	0.24	0.62	21	0.18	1.06	0.01	−25	49	0.29	1.01	5	0.06	0.35	0.01	−79
25–44	20	0.08	0.38	12	0.09	0.41	8	0.07	0.34	0.77	−22	17	0.10	0.43	3	0.03	0.23	0.77	−70
45–64	11	0.04	0.33	4	0.03	0.17	7	0.06	0.46	0.56	100	4	0.02	0.15	7	0.08	0.52	0.56	300
⩾65	9	0.03	0.25	6	0.04	0.29	3	0.03	0.21	0.54	−25	5	0.03	0.25	4	0.04	0.26	0.54	33

a Percent change = ((mean school holiday cases–mean school term cases)/mean school term cases)×100%.

**Fig. 4.**
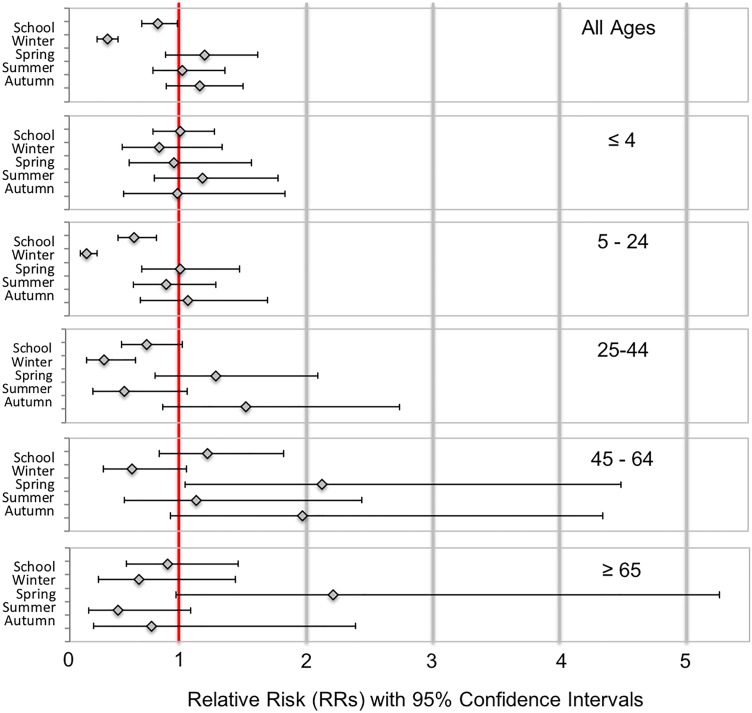
Estimates of relative risk (RR) with 95% confidence intervals for all university school holiday weeks (School) and individual school holiday weeks (Winter, Spring, Summer, Autumn) based on Model 3.

Negative binomial regression models' results support MW direct comparisons (see Supplementary Table S4). We sequentially constructed three models to determine the contribution of individual factors to weekly influenza tests. Model 1 is unadjusted and reflects the dampening or amplifying effects of either combined school holidays (marked as School) or individual school breaks (marked as Winter, Spring, Summer and Autumn). Model 2 was adjusted for average weekly dewpoint values, while Model 3 (shown in Fig. 4) was also adjusted for seasonal and linear trend. Modelling results indicate that School and Winter Break holiday weeks using both calendars had a dampening effect (RR < 1) in reported tests in all models for the all ages, 5–24 years and 25–44 years age groups. For the elderly, only university-based Summer Break was associated with a reduction in average weekly tests. For the youngest age group, only for the public-school Winter and Spring Breaks were associated with a reduction in average weekly tests. University-based Spring Break appeared to show an amplification of tests with RR ranging from 1.42 to 3.06 (Model 2); however, these associations did not hold for either calendar when we adjusted for seasonal and linear trends (Model 3). The discrepancies in observed effects across models were further explored by examining peak timing for influenza-specific outcomes.

### Peak timing estimation

**Fig. 5.**
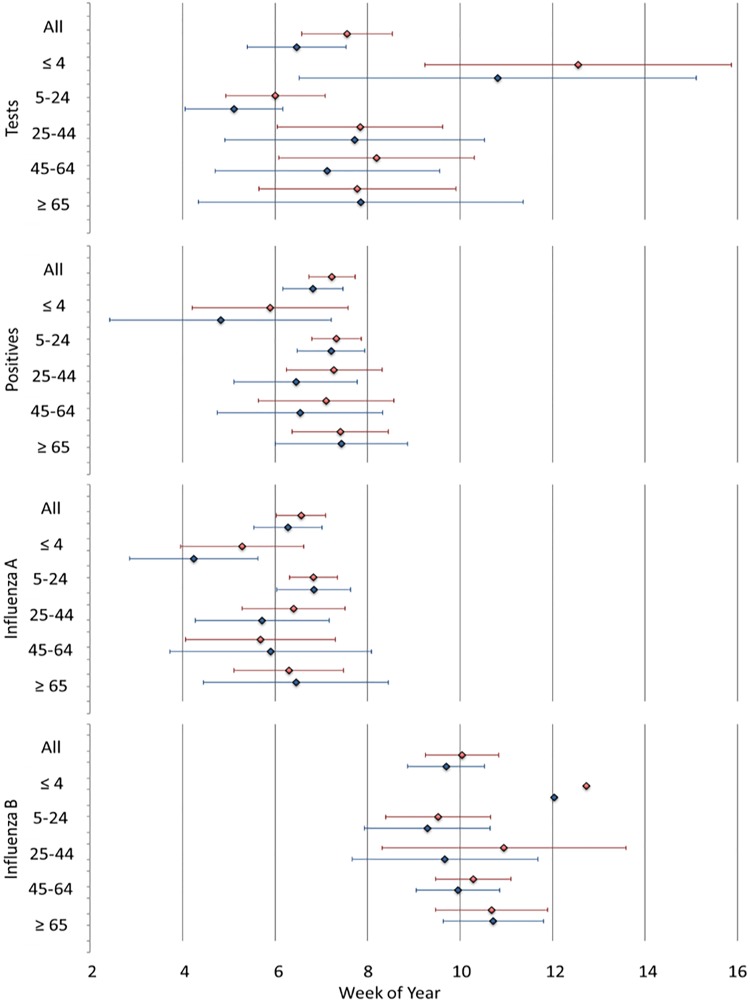
Estimates of peak timing for six age groups (all ages, ⩽4, 5–24, 25–44, 45–64, ⩾65 years) and four influenza outcomes (tests, positives, influenza A and influenza B) based on Model 4 (red) and Model 5 (blue).

We estimated peak timing and their confidence intervals for four outcomes and six age groups based on the 258-week study period using Models 3, 4 and 5 (see Fig. 5 and Supplementary Table S5). For all ages, influenza A peaked between the 6th and 7th calendar weeks right after the Winter Break and before the Spring Break. Influenza B peaked between the 8th and 12th calendar weeks right before or during the Spring Break. Based on Model 5, the peaks for influenza A preceded influenza B by 3.5 weeks for all ages (6.28, 95% CI 5.54–7.02 *vs.* 9.70, 95% CI 8.87–10.53 calendar week) and by 2.5 weeks for the 5–24 years age group (6.84, 95% CI 6.04–7.63 *vs.* 9.29, 95% CI 7.93–10.65 calendar week; Fig. 5). Although the differences between the estimates were not significant, peak timing obtained from Model 5 consistently preceded the estimates from Model 4. The peaks in influenza A and B typically coincide with the following events and observances: Super Bowl (6th calendar week), Ash Wednesday (6th–9th calendar weeks) and President's Day (8th calendar week).

### Winter and Spring Break holidays effects

**Fig. 6.**
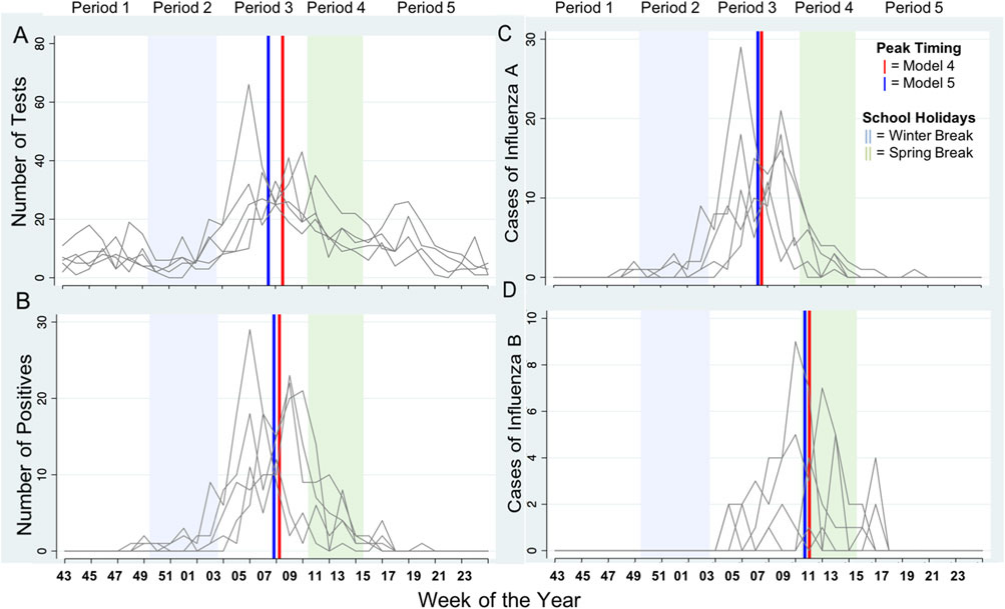
Weekly tests, positives, influenza A and B (panels A–D, respectively) for all ages across each study year with superimposed peak timing estimates in Milwaukee, WI (2004–2009). The five time periods include period 1 – before Winter Break (5 weeks), period 2 – during Winter Break (4–6 weeks), period 3 – between Winter and Spring Breaks (6–9 weeks), period 4 – during Spring Break (2–3 weeks) and period 5 – after Spring Break (5 weeks). All holiday breaks refer to the university school calendar.

Given the general similarity of university-based and public-school results, only university-based Winter and Spring holiday effects were investigated in greater details as described below. Examining influenza A and B more closely, there was a 4.8-fold decrease of influenza A during school holiday weeks (0.52 ± 1.63 *vs.* 2.49 ± 5.20, *P* < 0.005) for all ages, while average weekly counts of influenza B were near equivalent to school term weeks (0.33 ± 1.38 *vs.* 0.40 ± 1.02, *P* = 0.02; Table 2). As shown in Figure 6, we compared for all years five holiday periods: period 1 – before Winter Break (5 weeks), period 2 – during Winter Break (4–6 weeks), period 3 – between Winter and Spring Breaks (6–9 weeks), period 4 – during Spring Break (2–3 weeks) and period 5 – after Spring Break (5 weeks). The high counts of tests and positives fall either between the Winter and Spring Breaks (typically 2nd–9th calendar week) or during Spring Break alone (typically 10th–13th calendar week). Figure 6 displays the estimated peak timing in relation to the Winter and Spring Break school holiday periods.

Dampening of weekly tests, positives and influenza A during Winter Break is evident by significant differences in counts between periods 1 and 2 (3.60 ± 2.42 *vs.* 1.49 ± 1.53, *P* = 0.01) and between periods 2 and 3 (1.49 ± 1.53 *vs.* 14.23 ± 8.66, *P* < 0.005) for the 5–24 years age group (see Supplementary Table S6). Similarly, significant increases in tests (4.93 ± 2.81 *vs.* 22.12 ± 11.60, *P* < 0.005), positives (0.67 ± 0.82 *vs.* 9.61 ± 7.16, *P* < 0.005) and influenza A (0.67 ± 0.82 *vs.* 8.58 ± 6.77, *P* < 0.005) were observed after the Winter Break for all ages. No significant changes were detected for influenza B across any age group.

### Seasonal signatures

**Fig. 7.**
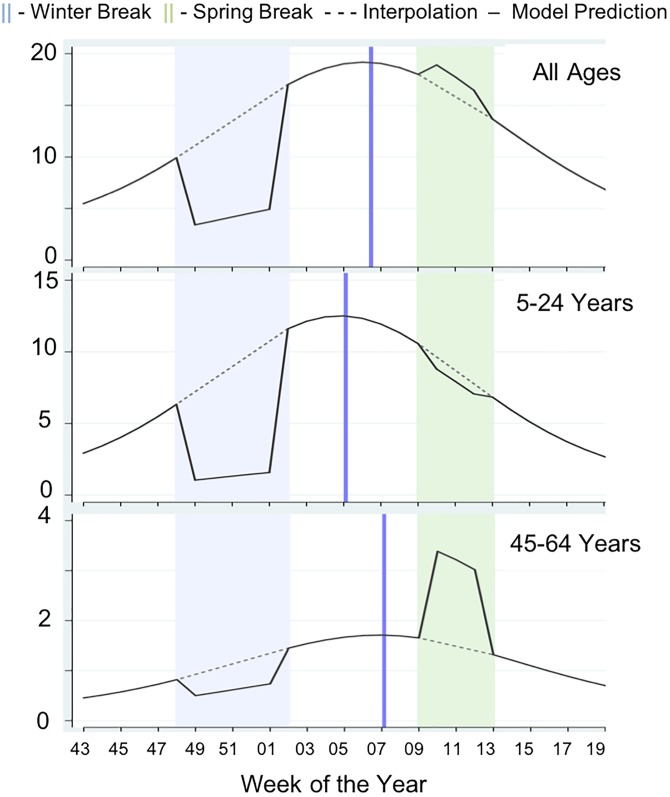
Seasonal signatures of weekly tests for time periods surrounding the Winter and Spring university school breaks for the all ages, 5–24 years and 45–64 years age groups (panels A, B and C, respectively).

In Figure 7, we plotted seasonal signatures of influenza incidence for time periods surrounding the Winter and Spring school breaks. Signatures were derived from Model 5 for all ages, 5–24 years and 45–64 years age groups across tests. The dampening of tests during Winter Break was evident in all ages and in those 5–24 years (RR = 0.31; 95% CI 0.22–0.41 *vs.* RR = 0.14; 95% CI 0.09–0.22, respectively). A significant increase in tests was observed during Spring Break in 45–64 years old adults (RR = 2.12; 95% CI 1.14–3.96).

### Effects of religious observances, federal holidays and sporting events

A direct comparison of average weekly tests, positives, influenza A and influenza B reported during weeks with Christian, Jewish or Muslim observances to those weeks without relevant observances show largely no significant differences (Supplementary Table S7). Similarly, no differences in four influenza-related outcomes were observed for federal holidays and sporting events (Supplementary Table S8). Overall, regression modelling results support the findings of direct comparisons for holiday categories (Supplementary Table S9). However, some discrepancies were noted for Christian observances.

Specifically, in the 25–44 years age group, there are increases in weekly tests (2.00 ± 1.83 *vs.* 1.07 ± 1.68, *P* = 0.01), positives (0.75 ± 0.86 *vs.* 0.31 ± 0.92, *P* < 0.005) and influenza A (0.75 ± 0.86 *vs.* 0.23 ± 0.80, *P* < 0.005), respectively (Supplementary Table S7). Similar patterns were observed for positives and influenza A for all ages though these observations did not hold in the fully adjusted regression model. The holiday amplification effect for tests in the 25–44 years age group most likely was driven by the alignment of Ash Wednesday to the peak timing for tests and positives. Based on regression modelling, average weekly tests for the 25–44 years age group during Ash Wednesday were significantly higher than weeks with no other Christian observances (RR = 2.56, 95% CI 1.55–4.21). At the same time, during Christmas weeks, which are aligned with Winter Break, a significant decrease in tests are detected by all models for the all ages and 5–24 years age groups (Supplementary Table S10).

In closer analyses of individual federal holidays and sporting events using regression models, we observed that President's Day showed significant increases in weekly tests for the all ages (RR = 1.41; 95% CI 1.10–1.82) and the 5–24 years (RR = 2.07; 95% CI 1.52–2.81) age groups. Similarly, the Super Bowl showed significant increases in weekly tests for the 5–24 years age group (RR = 1.79; 95% CI 1.15–2.77). These results confirmed direct comparisons shown in Supplementary Table S11. These two events are aligned with the period of high influenza incidence near the peak of influenza A.

## Discussion

We examined the impact of school holidays, religious observances and social events for six age groups (all ages, ⩽4, 5–24, 25–44, 45–64, ⩾65 years) on four health outcomes of influenza (total tested, positives, influenza A and B) in Milwaukee, WI from 2004 to 2009. Our analysis compared average weekly counts of health outcomes for holidays sharing a common theme (e.g. School, Christian, Jewish, Muslim, Federal and Sporting holidays) and individual holiday, observance or event periods. An increase in cases was most pronounced among holidays more closely aligned with the peak timing of positives, influenza A and influenza B health outcomes (ranging from 5th to 13th calendar week). In contrast, a decrease in cases was seen for holidays taking place during time periods of low incidence (ranging from 19th to 39th calendar week) or multiple weeks before and after the peak timing for influenza A and B. We observed amplification and dampening of cases during holidays that depended on proximity to seasonal peaks of influenza, most pronounced in school-aged children and young adults. These effects were consistent irrespective of using university-based or district-wide public-school holiday calendars.

Historical observations suggest that MU students have a large influence on the recorded influenza burden in the Greater Milwaukee area, potentially caused by the closeness of student-dwelling spaces (e.g. dormitories, cafeterias, etc.) and the centrality of the university with respect to much of the city's domestic and international transportation [32]. As 48% of tests and 65% of positives were attributable to the 5–24 years age group, in–out migration of Marquette students may be driving the dampening effects seen during the Winter Break holiday. City demographic characteristics from 2005 to 2009 show that 35.3% of Milwaukee residents were <25 years, 43.5% ranged from 25 to 54 years and 21.1% were >55 years (see Fig. 1). The large proportion of younger- and older-adult residents along with the MU student population likely contribute to the significant holiday effects for the 5–24, 25–44 and 45–64 years age groups [33]. High migration out of the city by MU students during Winter Break is likely to reduce the child-to-child, child-to-adult and adult-to-adult transmission patterns described within other works [3, 7, 12]. Similarly, student migration out of Milwaukee during Spring Break may support significantly reduced influenza B positives during these calendar weeks. Contrastingly, the return from the school holiday breaks is likely to increase the exchange and introduction of the virus to the community.

To the best of our knowledge, this is one of the few studies using time series analysis to examine seasonal signatures of individual holiday effects. All reviewed works primarily evaluated transmission patterns of influenza during extreme mass gatherings [20, 21, 34]. Otherwise, researchers rarely considered differential effects of holidays at different times of the year, instead combining all holiday weeks under one dichotomous variable [13] or isolating their analyses to a single time of the year [35]. While some works have reviewed differences in incidence risk ratios (IRRs) before, during and after individual school holidays, none have explored holiday categorisation effects on these IRRs or seasonal signatures [2, 3, 6]. Therefore, our results serve to: (1) confirm dampening of influenza incidence during wintertime school holidays seen using both academic calendars, (2) demonstrate how holiday categorisation effects suspected amplification or dampening of incidence in time series modelling, and (3) illustrate how seasonal signature estimates of peak timing can be modified by holiday inclusion when modelling.

Though much smaller in scope, our findings showed significant amplification of cases during springtime holiday weeks (Ash Wednesday, Easter and the Prophet's Birthday) compared to non-holiday weeks using both non-parametric and log-linear regression analyses. The observed associations with the Christian holidays in Milwaukee County are supported by over 94% of religious-goers belonging to a Christian or Catholic congregation [27]. According to the 2010 Census, approximately 439 526 (46.4%) residents of the total population affiliated to any religious group; among those, 45.31% residents identified themselves as Catholic, 46.99% – Protestant, 2.07% – Orthodox Christian, 1.73% – Jewish, 2.08% – Muslim and 1.82% other religious affiliation. The studied religious observances are not exhaustive. A significant increase in Muslim adherents, as well as unchanging levels of Jewish adherents from 2000 to 2010, justifies further analyses of these religious holidays. Furthermore, the significant outnumbering of Christian followers compared to all other religious groups also justified further investigation of Christian holiday-related effects. However, as these holidays are often short (1–4 days), time series analyses are susceptible to surveillance reporting delays. Future research over a longer time series and in a larger population is needed to investigate more detailed seasonal signatures for these holidays.

Our results show that while holidays may be clustered close together within the high-incidence influenza season, age-specific peak timing variations among health outcomes can result in dissimilar effects. On the one hand, School Break holidays exemplify how peak timing of certain health outcomes may consistently align with some holidays more than others, irrespective of age groups. This alignment explains the consistent amplification during Spring Break compared to the dampening effects of Winter Break. We demonstrated that the collective categorisation of holidays, such as School Break weeks, can mask the individual effect of one holiday (in our case – Spring Break) due to the near-zero incidence of other individual holidays within that category (e.g. Summer and Autumn Breaks). In addition, when holidays cluster near one another, they may manifest similar effects with respect to age-specific health outcomes. This was observed for Ash Wednesday, President's Day and the Super Bowl, which fell between the 6th and 10th calendar weeks and aligned closely to influenza A peak timing for school-aged children and young adults. Alternatively, the slightly delayed occurrence of Spring Break, Easter and the Prophet's Birthday aligned more closely with influenza B peak timing for the all ages and 45–64 years age groups, a pattern not shared by the three earlier-occurring holidays.

These differences in observed effects despite small time intervals between holidays' occurrences exemplify the importance of age- and outcome-specific peak timing analyses. Crude aggregation of influenza cases by broad age categories is likely to mask nuanced relationships between influenza incidence and commonly occurring holidays at the sub-population level. In this study, we oversampled the college-aged population. Per the location of the sampling site and per the scope of this study, most of the collected samples were derived from patients and students attending MU, located in downtown Milwaukee. The laboratory is one of the surveillance sites in Milwaukee jurisdiction, and is likely to capture the majority of flu cases in primary zip codes for the MU campus and student-preferred residencies (53233, 53202 and 53203). The urban campus also allows off campus residence, so students were also able to be part of the urban setting while still attending college. As the study oversampled MU students, the sampling ages within the WHO bracket representing 5–24 years old are likely to be closer to the 18–24 range (college age groups). Our inability to disaggregate the 5–24 years age group into younger- and older-age school children remains a limitation we could not adjust for. This standard reporting practice prevents investigating differential effects for Milwaukee public-school students (elementary to high school, 5–18 years) and MU students (19–24 years). While we applied two academic calendars and observed the holiday effects with both calendars, a more detailed age breakdown would have provided more accurate estimates and offered further insight into how school holiday-related events may impact influenza transmission during those periods.

The observed holiday effects were detected with both academic calendars, yet there were differences in Spring Break timing: amplification effects were seen during Spring Break for university school breaks, occurring typically in March and during public-school breaks about 2 weeks later. University Summer Breaks typically began 1–2 weeks before the public-school district, while Winter Breaks began 1 week earlier and lasted 1 week later. Spring Breaks were typically 2 weeks apart between academic calendars. The public-school district had no annual Autumn Break (except 2008). As all public schools followed a single district-wide academic calendar, we were not able to explore individual differences between primary and secondary or public and private schools. As we choose the undergraduate full-time student calendars with four consistently non-overlapping school holidays (i.e. Winter, Spring, Summer and Autumn Breaks) to represent school holidays for the entire 5–24 years age group, it is likely that the estimates of the observed effects are conservative. A marginal increase in the effects detected by the public-school calendar supports this assertion. A future analysis of more refined age groups aligned with their respective school calendars is needed to verify our findings.

Holiday effects are not easily deciphered for overlapping holidays. While holidays within each combined holiday category have little overlap, holidays between categories clustered at specific times of the year. As such, risk ratios may have been confounded by the effect of a combination of coinciding holidays as opposed to a single holiday event. As such, dampening or amplification effects may capture the synergy of collective holiday effects; the overlapping holidays can either attenuate towards no effect or falsely magnify a minimal effect. Our analysis accounts for this by analysing both individual and collective holiday effects and by building a model that accounts for seasonal oscillations. Furthermore, the consistency of both average weekly cases and risk ratios from regression models confirms the isolated effect of a single holiday in our study.

Holiday effects can be also amplified by meteorological conditions. To provide some robustness to the model, we incorporated dewpoint values representing the perceived ambient temperature corrected for the air moisture content to all modelling procedures. In general, cold and dry conditions may enhance the transmission and survival of the influenza virus or other respiratory pathogens. Many studies had demonstrated an increased risk for influenza and pneumonia associated with the low temperature and humidity [36–39]. We also detected an independent effect of the weather-related parameter even after controlling for influenza seasonality. However, we did not explore the joint effect of school closures related to extreme weather, which would require a longer time series to capture a sufficient number of rare events.

Even without overlapping weeks, clustered holidays are also liable to effect modification from surveillance system reporting delays of cases for holidays spanning multiple weeks. This results in a misalignment between weeks with assigned holidays and weeks with reported cases. For example, if a holiday occurs at the end of the week and the MHDL is closed due to a holiday, influenza surveillance would not continue until the following Monday. Cases originating due to transmission during the prior week would now be reported the following week. Even in the absence of a reporting delay, the multi-day incubation period of influenza may create this misalignment for short-duration holiday events. We accounted for this through examinations of seasonal signatures for Winter and Spring Break holiday effects though not elsewhere within our analyses. A simple approach of comparing risks of respiratory infections within communities while schools were in session and out-of-session is a viable approach to start [40], yet the complexity of accounting for overlapping events and delayed effects is daunting.

The conducted analysis shows the extreme care researchers must take when defining holiday-related variables and considering their inclusion in time series modelling. If all holiday weeks are combined into a single, collective category, researchers will likely overestimate the protective effect these holidays play in mitigating influenza incidence. Thus, careful analyses of age- and outcome-specific peak timing can help to inform modellers what surrounding holidays may be influential in detecting dampening or amplifying effects. Although the isolation of individual holidays during periods of expected high influenza incidence can be extremely laborious, a well-defined and consistent effect can be properly considered as a controllable risk factor for the analysis of influenza seasonality and improve influenza forecasting. While full-scale randomised control studies to measure the effect of school closures as prevention strategies are desirable, such studies might be prohibitively expensive. Joint efforts of local health departments to share data for an inclusive analysis would allow to expand the scope of the presented study.

## Conclusion

Our study emphasises the importance of understanding influenza seasonality, or periodic behaviour of incidence over time, when exploring associations between calendar effects and disease outcomes. We applied time series analyses – methods performed on time-referenced data to describe, explain and predict temporal dynamics – to analyse seasonality and its important feature such as peak timing [41]. The applied time series analyses allowed us to formally compare seasonality characteristics across case definitions and age groups and to detect differential effects of calendar events. These effects were detected in publicly available records maintained by routine influenza surveillance. Our results show that holiday effects are largely contingent upon the alignment of that holiday in relation to incidence peak timing and sensitive to how one defines the holiday (collective or individual holiday weeks). Combining individual holidays into collective holiday periods potentially leads to misspecification by masking individual holiday effects. These effects are differential across age and health outcomes, requiring researchers to pay particular attention to holiday-peak timing relationships within a modelling framework. We recommend incorporating location-specific calendar effects in influenza modelling and near-term forecasts tailored to susceptible age groups to better predict and assess targeted intervention measures.

## Supplementary material

For supplementary material accompanying this paper visit https://doi.org/10.1017/S0950268819001511. click here to view supplementary material
