# Supplementary material for: Incorporating calendar effects to predict influenza seasonality in Milwaukee, Wisconsin
Source: Epidemiol Infect. 2019 Sep 11;147:e268. doi: 10.1017/S0950268819001511 (PMC6805754; doi:10.1017/S0950268819001511)
Supplement: Supplementary file 1 [file S0950268819001511sup001.docx]

**Epidemiology and Infection**

**Incorporating calendar effects to forecast influenza seasonality: A case study in Milwaukee, Wisconsin**

Ryan B. Simpson^1^, Tania M. Alarcon Falconi^1^, Aishwarya Venkat^1^, Kenneth H.H. Chui^1^, Jose Navidad^3^, Yuri N. Naumov^2^, Jack Gorski^2^, Sanjib Bhattacharyya^3^, Elena N. Naumova^1^

1. Friedman School of Nutrition Science and Policy, Tufts University, Boston, MA, United States.

2. Blood Research Center, Milwaukee, WI, United States.

3. City of Milwaukee Health Department Laboratory (MHDL), Milwaukee, WI, United States.

**Running Head:**

Calendar Effects and Influenza

**Supplemental Material**

Supplemental Table S1. Durations by day-of-year and week-of-year of the selected school holidays, national and religious observances, and sporting events during the study period (2004-2009) in Milwaukee, WI.

| Holiday | 2004 | 2005 | 2006 | 2007 | 2008 | 2009 |
| --- | --- | --- | --- | --- | --- | --- |
| Milwaukee Public School District Holidays | | | | | | |
| Winter ^a, c^ | 17-Dec - 3-Jan  50 – 1 | 21-Dec - 3-Jan  51 – 1 | 22-Dec - 3-Jan  51 – 1 | 21-Dec - 3-Jan  51 – 1 | 19-Dec - 5-Jan  51 – 1 | - |
| Spring ^b^ | - | 24-Mar - 4-Apr  12 – 13 | 13-Apr - 24-Apr  15 – 16 | 5-Apr - 16-Apr  14 – 15 | 20-Mar - 31-Mar  12 – 13 | 9-Apr - 20-Apr  15 – 16 |
| Summer | 15-Jun - 1-Sep  25 – 36 | 15-Jun - 1-Sep  24 – 35 | 13-Jun - 5-Sep  24 – 36 | 14-Jun - 4-Sep  24 – 36 | 12-Jun - 2-Sep  24 – 36 | - |
| Marquette University School Holidays | | | | | | |
| Winter ^a, c^ | 24-Dec - 8-Jan | 17-Dec - 14-Jan | 10-Dec - 14-Jan | 12-Dec - 7-Jan | 7-Dec - 17-Jan | - |
|  | 51 – 1 | 50 – 2 | 50 – 2 | 50 – 2 | 50 – 3 |  |
| Spring ^b^ | - | 13-Mar - 2-Apr | 5-Mar - 25-Mar | 11-Mar - 24-Mar | 9-Mar - 29-Mar | 1-Mar - 21-Mar |
|  |  | 11 – 13 | 10 – 12 | 11 – 12 | 11 – 13 | 10 – 12 |
| Summer | 6-Jun - 4-Sep | 29-May - 3-Sep | 28-May - 2-Sep | 27-May - 1-Sep | 25-May - 30-Aug | - |
|  | 24 – 36 | 22 – 35 | 22 – 35 | 22 – 35 | 22 – 35 |  |
| Autumn ^b, c^ | 17-Oct - 30-Oct | 16-Oct - 29-Oct | 15-Oct - 28-Oct | 14-Oct - 27-Oct | 12-Oct - 25-Oct | - |
|  | 43 – 44 | 42 – 43 | 42 – 43 | 42 – 43 | 42 – 43 |  |
| Christian Holidays | | | | | | |
| Ash  Wednesday ^b^ | - | 9-Feb | 1-Mar | 21-Feb | 6-Feb | 25-Feb |
|  |  | 6 | 9 | 8 | 6 | 9 |
| Easter ^b^ | - | 27-Mar - 28-Mar | 16-Apr - 17-Apr | 8-Apr - 9-Apr | 23-Mar - 24-Mar | 12-Apr -13-Apr |
|  |  | 13 | 16 | 15 | 13 | 16 |
| Christmas ^c^ | 25-Dec | 25-Dec | 25-Dec | 25-Dec | 25-Dec | - |
|  | 52 | 51 | 52 | 52 | 52 |  |
| Jewish Holidays | | | | | | |
| Passover ^b^ | - | 24-Apr - 1-May | 13-Apr - 20-Apr | 3-Apr - 10-Apr | 20-Apr - 27-Apr | 9-Apr - 16-Apr |
|  |  | 17 – 18 | 15 – 16 | 14 – 15 | 17 – 18 | 15 – 16 |
| Rosh Hashanah ^c^ | 16-Sep | 4-Oct | 23-Sep | 13-Sep | 30-Sep | - |
|  | 38 | 38 | 40 | 38 | 37 |  |
| Yom Kippur ^c^ | 25-Sep | 13-Oct | 2-Oct | 22-Sep | 9-Oct | - |
|  | 39 | 41 | 40 | 38 | 41 |  |
| Sukkot ^c^ | 6-Oct | 24-Oct | 13-Oct | 3-Oct | 20-Oct | - |
|  | 41 | 43 | 41 | 40 | 43 |  |
| Hanukkah ^a, c^ | 8-Dec - 15-Dec | 26-Dec - 2-Jan | 16-Dec - 23-Dec | 5-Dec - 12-Dec | 22-Dec - 29-Dec | - |
|  | 50 – 51 | 52 – 1 | 50 – 51 | 49 – 50 | 52 – 1 |  |
| Muslim Holidays | | | | | | |
| Eid al-Adha ^b, c^ | - | 21-Jan | 10-Jan & 31-Dec | 20-Dec | 9-Dec | - |
|  |  | 3 | 2 & 52 | 51 | 50 |  |
| Muharram ^b, c^ | - | 10-Feb | 31-Jan | 20-Jan | 10-Jan, 29-Dec | - |
|  |  | 6 | 5 | 3 | 2 & 52 |  |
| Prophet’s Birthday ^b, c^ | - | 21-Apr | 11-Apr | 31-Mar | 20-Mar | - |
|  |  | 16 | 15 | 13 | 12 |  |
| Isra and Mi’raj ^c^ | 12-Sep | 1-Sep | 22-Aug | 11-Aug | 31-Jul | - |
|  | 38 | 35 | 34 | 32 | 31 |  |
| Ramadan ^c^ | 16-Oct - 14-Nov | 5-Oct - 4-Nov | 24-Sep - 24-Oct | 13-Sep - 13-Oct | 2-Sep - 2-Oct | - |
|  | 42 – 47 | 40 – 44 | 39 – 43 | 37 – 41 | 36 – 40 |  |
| Lailat al-Qadr ^c^ | 10-Nov | 30-Oct | 19-Oct | 8-Oct | 27-Sep | - |
|  | 46 | 44 | 42 | 41 | 39 |  |

Supplemental Table S1 continues onto the following page

| Federal Observances | | | | | | |
| --- | --- | --- | --- | --- | --- | --- |
| New Years ^b^ | - | 1-Jan | 1-Jan | 1-Jan | 1-Jan | 1-Jan |
|  |  | 1 | 1 | 1 | 1 | 1 |
| MLK Jr. Day ^b^ | - | 17-Jan | 16-Jan | 15-Jan | 21-Jan | 19-Jan |
|  |  | 3 | 3 | 3 | 4 | 4 |
| President’s Day ^b^ | - | 21-Feb | 20-Feb | 19-Feb | 18-Feb | 16-Feb |
|  |  | 8 | 8 | 8 | 8 | 8 |
| Memorial Day ^c^ | 31-May | 30-May | 29-May | 28-May | 26-May | - |
|  | 22 | 22 | 22 | 22 | 22 |  |
| Independence Day ^c^ | 4-Jul | 4-Jul | 4-Jul | 4-Jul | 4-Jul | - |
|  | 27 | 27 | 27 | 27 | 27 |  |
| Labor Day ^c^ | 6-Sep | 5-Sep | 4-Sep | 3-Sep | 1-Sep | - |
|  | 36 | 36 | 36 | 36 | 36 |  |
| Columbus  Day ^c^ | 11-Oct | 10-Oct | 9-Oct | 8-Oct | 13-Oct | - |
|  | 42 | 41 | 41 | 41 | 42 |  |
| Veteran’s Day ^c^ | 11-Nov | 11-Nov | 11-Nov | 11-Nov | 11-Nov | - |
|  | 46 | 45 | 45 | 46 | 46 |  |
| Thanksgiving ^c^ | 25-Nov | 24-Nov | 23-Nov | 22-Nov | 27-Nov | - |
|  | 48 | 47 | 47 | 47 | 48 |  |
| Sporting Events | | | | | | |
| Super Bowl ^b^ | - | 6-Feb | 5-Feb | 4-Feb | 3-Feb | 1-Feb |
|  |  | 6 | 6 | 6 | 6 | 6 |
| Triple Crown ^c^ | 5 Jun | 7-May, 21-May, & 11-Jun | 6-May, 20-May, & 10-Jun | 5-May, 19-May, & 9-Jun | 3-May, 17-May, & 7-Jun | - |
|  | 23 | 18, 20, 23 | 18, 20, 23 | 18, 20, 23 | 18, 20, 23 |  |
| NHL Finals ^c^ | 25-May - 7-Jun | - ^d^ | 5-Jun - 19-Jun | 28-May - 6-Jun | 24-May - 4-Jun | - |
|  | 22 – 24 |  | 23 – 25 | 22 – 23 | 21 – 23 |  |
| NBA Finals ^c^ | 6-Jun - 15-Jun | 9-Jun - 23-Jun | 8-Jun - 20-Jun | 7-Jun - 14-Jun | 5-Jun - 17-Jun | - |
|  | 24 – 25 | 23 – 25 | 23 – 25 | 23 – 24 | 23 – 25 |  |
| World Series ^c^ | 23-Oct - 27-Oct | 22-Oct - 26-Oct | 21-Oct - 27-Oct | 24-Oct - 28-Oct | 22-Oct - 29-Oct | - |
|  | 43 – 44 | 42 – 43 | 42 – 43 | 43 – 44 | 43 – 44 |  |

^a^ Holidays weeks that span multiple years.

^b^ Holidays that, in 2004, occurred prior to the 258-week study period.

^c^ Holidays that, in 2009, occurred after the 258-week study period.

^d^ In 2005, the National Hockey League was on strike so no championship finals was played.

Supplemental Table S2. Summary statistics for four influenza health outcomes (tests, positives, influenza A, and influenza B) and six age groups (all ages, ≤ 4, 5-24, 25-44, 45-64, and ≥ 65 years) across each calendar year and the full study period (2004-2009) in Milwaukee, WI.

| Year | Tests | | | | | Positives | | | | | Influenza A | | | | | Influenza B | | | | |
| --- | --- | --- | --- | --- | --- | --- | --- | --- | --- | --- | --- | --- | --- | --- | --- | --- | --- | --- | --- | --- |
|  | Count | Mean | SD | Skew* | Kurt* | Count | Mean | SD | Skew* | Kurt* | Count | Mean | SD | Skew* | Kurt* | Count | Mean | SD | Skew* | Kurt* |
| All Ages | | | | | | | | | | | | | | | | | | | | |
| 2004 | 250 | 7.58 | 4.94 | 0.59 | 2.59 | 3 | 0.09 | 0.38 | 4.30 | 20.63 | 3 | 0.09 | 0.38 | 4.30 | 20.63 | 0 | 0.00 | 0.00 | . | . |
| 2005 | 513 | 9.87 | 11.63 | 2.89 | 12.74 | 104 | 2.00 | 5.46 | 3.46 | 15.08 | 100 | 1.92 | 5.44 | 3.50 | 15.38 | 4 | 0.08 | 0.33 | 4.60 | 24.28 |
| 2006 | 502 | 9.65 | 7.59 | 1.09 | 3.49 | 56 | 1.08 | 2.54 | 3.02 | 12.02 | 46 | 0.88 | 2.44 | 3.47 | 14.91 | 10 | 0.19 | 0.66 | 4.37 | 23.75 |
| 2007 | 383 | 7.37 | 7.01 | 1.53 | 4.51 | 103 | 1.98 | 4.41 | 2.89 | 12.10 | 96 | 1.85 | 4.08 | 2.83 | 11.64 | 7 | 0.13 | 0.44 | 3.35 | 13.32 |
| 2008 | 459 | 8.83 | 10.33 | 1.36 | 4.01 | 127 | 2.44 | 5.01 | 2.29 | 7.72 | 92 | 1.77 | 4.10 | 2.82 | 10.60 | 35 | 0.67 | 1.59 | 2.43 | 8.06 |
| 2009 | 271 | 15.94 | 12.03 | 0.92 | 2.79 | 112 | 6.59 | 7.83 | 0.78 | 2.01 | 73 | 4.29 | 5.91 | 1.02 | 2.39 | 39 | 2.29 | 2.73 | 1.10 | 3.31 |
| Total | 2378 | 9.22 | 9.24 | 2.11 | 9.48 | 505 | 1.96 | 4.68 | 3.04 | 12.58 | 410 | 1.59 | 4.10 | 3.43 | 16.16 | 95 | 0.37 | 1.20 | 4.18 | 22.56 |
| ≤4 Years | | | | | | | | | | | | | | | | | | | | |
| 2004 | 69 | 2.09 | 2.01 | 0.72 | 2.55 | 0 | 0.00 | 0.00 | . | . | 0 | 0.00 | 0.00 | . | . | 0 | 0.00 | 0.00 | . | . |
| 2005 | 114 | 2.19 | 2.32 | 1.20 | 3.96 | 4 | 0.08 | 0.33 | 4.60 | 24.28 | 4 | 0.08 | 0.33 | 4.60 | 24.28 | 0 | 0.00 | 0.00 | . | . |
| 2006 | 135 | 2.60 | 2.01 | 0.17 | 2.33 | 3 | 0.06 | 0.24 | 3.79 | 15.39 | 2 | 0.04 | 0.19 | 4.80 | 24.04 | 1 | 0.02 | 0.14 | 7.00 | 50.02 |
| 2007 | 97 | 1.87 | 1.86 | 0.77 | 2.81 | 3 | 0.06 | 0.24 | 3.79 | 15.39 | 3 | 0.06 | 0.24 | 3.79 | 15.39 | 0 | 0.00 | 0.00 | . | . |
| 2008 | 78 | 1.50 | 1.91 | 0.96 | 2.94 | 1 | 0.02 | 0.14 | 7.00 | 50.02 | 1 | 0.02 | 0.14 | 7.00 | 50.02 | 0 | 0.00 | 0.00 | . | . |
| 2009 | 34 | 2.00 | 2.45 | 1.08 | 3.25 | 1 | 0.06 | 0.24 | 3.75 | 15.06 | 1 | 0.06 | 0.24 | 3.75 | 15.06 | 0 | 0.00 | 0.00 | . | . |
| Total | 527 | 2.04 | 2.07 | 0.84 | 3.14 | 12 | 0.05 | 0.23 | 5.25 | 32.60 | 11 | 0.04 | 0.22 | 5.57 | 36.54 | 1 | 0.00 | 0.06 | 15.97 | 256.0 |
| 5-24 Years | | | | | | | | | | | | | | | | | | | | |
| 2004 | 91 | 2.76 | 3.26 | 1.23 | 3.27 | 1 | 0.03 | 0.17 | 5.48 | 31.03 | 1 | 0.03 | 0.17 | 5.48 | 31.03 | 0 | 0.00 | 0.00 | . | . |
| 2005 | 252 | 4.85 | 7.09 | 2.53 | 9.24 | 54 | 1.04 | 2.84 | 2.98 | 11.02 | 52 | 1.00 | 2.84 | 3.02 | 11.18 | 2 | 0.04 | 0.28 | 7.00 | 50.02 |
| 2006 | 219 | 4.21 | 4.24 | 1.37 | 4.25 | 32 | 0.62 | 1.55 | 3.16 | 12.50 | 26 | 0.50 | 1.48 | 3.64 | 15.73 | 6 | 0.12 | 0.58 | 6.00 | 39.74 |
| 2007 | 225 | 4.33 | 5.94 | 2.21 | 7.08 | 76 | 1.46 | 3.83 | 3.69 | 18.15 | 69 | 1.33 | 3.50 | 3.68 | 17.93 | 7 | 0.13 | 0.44 | 3.35 | 13.32 |
| 2008 | 186 | 3.58 | 5.80 | 2.42 | 8.22 | 70 | 1.35 | 3.30 | 2.83 | 10.03 | 62 | 1.19 | 3.09 | 3.03 | 11.28 | 8 | 0.15 | 0.41 | 2.71 | 9.95 |
| 2009 | 180 | 10.59 | 9.68 | 0.75 | 2.27 | 93 | 5.47 | 6.81 | 0.94 | 2.35 | 62 | 3.65 | 5.04 | 1.13 | 2.87 | 31 | 1.82 | 2.46 | 1.75 | 5.55 |
| Total | 1153 | 4.47 | 6.12 | 2.32 | 8.49 | 326 | 1.26 | 3.39 | 3.48 | 15.87 | 272 | 1.05 | 2.93 | 3.50 | 16.04 | 54 | 0.21 | 0.85 | 6.54 | 56.81 |
| 25-44 Years | | | | | | | | | | | | | | | | | | | | |
| 2004 | 30 | 0.91 | 1.21 | 1.25 | 3.60 | 1 | 0.03 | 0.17 | 5.48 | 31.03 | 1 | 0.03 | 0.17 | 5.48 | 31.03 | 0 | 0.00 | 0.00 | . | . |
| 2005 | 62 | 1.19 | 1.62 | 2.70 | 12.55 | 17 | 0.33 | 0.94 | 3.69 | 16.83 | 15 | 0.29 | 0.91 | 4.06 | 19.54 | 2 | 0.04 | 0.19 | 4.80 | 24.04 |
| 2006 | 78 | 1.50 | 2.19 | 2.00 | 7.03 | 10 | 0.19 | 0.74 | 4.04 | 18.70 | 7 | 0.13 | 0.69 | 4.96 | 26.12 | 3 | 0.06 | 0.31 | 5.54 | 33.52 |
| 2007 | 42 | 0.81 | 1.24 | 2.00 | 7.73 | 21 | 0.40 | 0.87 | 2.38 | 8.43 | 21 | 0.40 | 0.87 | 2.38 | 8.43 | 0 | 0.00 | 0.00 | . | . |
| 2008 | 51 | 0.98 | 1.66 | 1.94 | 6.05 | 24 | 0.46 | 1.00 | 2.07 | 6.13 | 13 | 0.25 | 0.62 | 2.74 | 10.40 | 11 | 0.21 | 0.61 | 2.56 | 7.70 |
| 2009 | 28 | 1.65 | 2.21 | 1.90 | 5.69 | 14 | 0.82 | 1.70 | 2.16 | 6.51 | 10 | 0.59 | 1.50 | 3.02 | 11.25 | 4 | 0.24 | 0.75 | 3.23 | 12.18 |
| Total | 291 | 1.13 | 1.70 | 2.32 | 9.43 | 87 | 0.34 | 0.92 | 3.31 | 14.67 | 67 | 0.26 | 0.81 | 4.12 | 22.21 | 20 | 0.08 | 0.38 | 5.21 | 30.79 |
| 45-64 Years | | | | | | | | | | | | | | | | | | | | |
| 2004 | 26 | 0.79 | 1.02 | 1.32 | 4.34 | 0 | 0.00 | 0.00 | . | . | 0 | 0.00 | 0.00 | . | . | 0 | 0.00 | 0.00 | . | . |
| 2005 | 41 | 0.79 | 0.89 | 0.93 | 3.02 | 7 | 0.13 | 0.44 | 3.35 | 13.32 | 7 | 0.13 | 0.44 | 3.35 | 13.32 | 0 | 0.00 | 0.00 | . | . |
| 2006 | 37 | 0.71 | 1.04 | 1.88 | 7.26 | 4 | 0.08 | 0.27 | 3.18 | 11.08 | 4 | 0.08 | 0.27 | 3.18 | 11.08 | 0 | 0.00 | 0.00 | . | . |
| 2007 | 13 | 0.25 | 0.65 | 2.69 | 9.54 | 2 | 0.04 | 0.19 | 4.80 | 24.04 | 2 | 0.04 | 0.19 | 4.80 | 24.04 | 0 | 0.00 | 0.00 | . | . |
| 2008 | 90 | 1.73 | 3.07 | 2.51 | 8.83 | 17 | 0.33 | 1.08 | 3.57 | 14.92 | 8 | 0.15 | 0.64 | 4.89 | 27.91 | 9 | 0.17 | 0.71 | 4.47 | 22.44 |
| 2009 | 20 | 1.18 | 1.07 | 0.27 | 1.79 | 2 | 0.12 | 0.33 | 2.37 | 6.63 | 0 | 0.00 | 0.00 | . | . | 2 | 0.12 | 0.33 | 2.37 | 6.63 |
| Total | 227 | 0.88 | 1.66 | 4.29 | 27.77 | 32 | 0.12 | 0.56 | 6.37 | 50.10 | 21 | 0.08 | 0.38 | 6.33 | 52.74 | 11 | 0.04 | 0.33 | 9.63 | 103.04 |
| ≥65 Years | | | | | | | | | | | | | | | | | | | | |
| 2004 | 33 | 1.00 | 1.20 | 0.88 | 2.59 | 1 | 0.03 | 0.17 | 5.48 | 31.03 | 1 | 0.03 | 0.17 | 5.48 | 31.03 | 0 | 0.00 | 0.00 | . | . |
| 2005 | 44 | 0.85 | 2.80 | 5.62 | 36.00 | 22 | 0.42 | 2.02 | 6.10 | 40.77 | 22 | 0.42 | 2.02 | 6.10 | 40.77 | 0 | 0.00 | 0.00 | . | . |
| 2006 | 34 | 0.65 | 1.27 | 2.25 | 8.22 | 7 | 0.13 | 0.49 | 4.48 | 24.92 | 7 | 0.13 | 0.49 | 4.48 | 24.92 | 0 | 0.00 | 0.00 | . | . |
| 2007 | 6 | 0.12 | 0.47 | 4.93 | 28.91 | 1 | 0.02 | 0.14 | 7.00 | 50.02 | 1 | 0.02 | 0.14 | 7.00 | 50.02 | 0 | 0.00 | 0.00 | . | . |
| 2008 | 53 | 1.02 | 2.01 | 2.15 | 6.49 | 14 | 0.27 | 0.93 | 4.88 | 29.18 | 7 | 0.13 | 0.53 | 4.27 | 21.22 | 7 | 0.13 | 0.53 | 4.27 | 21.22 |
| 2009 | 8 | 0.47 | 0.62 | 0.91 | 2.81 | 2 | 0.12 | 0.33 | 2.37 | 6.63 | 0 | 0.00 | 0.00 | . | . | 2 | 0.12 | 0.33 | 2.37 | 6.63 |
| Total | 178 | 0.69 | 1.74 | 5.76 | 51.84 | 47 | 0.18 | 1.03 | 10.38 | 129.88 | 38 | 0.15 | 0.97 | 11.83 | 163.3 | 9 | 0.03 | 0.25 | 8.78 | 88.66 |

* “Skew” is an abbreviation for skewness while “Kurt” is an abbreviation for kurtosis

Supplemental Table S3. Influenza outcome-specific counts and weekly averages with standard deviations for all ages by individual holiday according to CDC-defined high-, moderate- and low-incidence periods [32].

| Holiday | Total Cases* | | | | Average Weekly Cases | | | |
| --- | --- | --- | --- | --- | --- | --- | --- | --- |
|  | Tests | Positives | Influenza A | Influenza B | Tests | Positives | Influenza A | Influenza B |
| High Incidence period | | | | | | | | |
| Winter (Uni.)**  Winter (Public)** | 120  70 | 16  10 | 16  10 | 0  0 | 4.80 (3.03)  4.38 (3.40) | 0.64 (0.86)  0.63 (0.89) | 0.64 (0.86)  0.63 (0.89) | -  - |
| Ash Wednesday | 151 | 71 | 66 | 5 | 30.20 (5.76) | 14.20 (6.42) | 13.20 (5.89) | 1.00 (1.73) |
| Christmas | 18 | 3 | 3 | 0 | 3.00 (2.10) | 0.50 (0.55) | 0.50 (0.55) | - |
| Hanukkah | 59 | 6 | 6 | 0 | 5.90 (5.22) | 0.60 (1.07) | 0.60 (1.07) | - |
| Eid al-Adha | 38 | 7 | 7 | 0 | 7.60 (5.94) | 1.40 (2.61) | 1.40 (2.61) | - |
| Muharram | 55 | 22 | 22 | 0 | 11.00 (15.44) | 4.40 (7.64) | 4.40 (7.64) | - |
| New Years | 33 | 5 | 5 | 0 | 6.60 (5.03) | 1.00 (1.41) | 1.00 (1.41) | - |
| MLK Jr. Day | 61 | 16 | 16 | 0 | 12.20 (6.57) | 3.20 (3.65) | 3.20 (3.65) | - |
| President’s Day | 128 | 54 | 47 | 7 | 25.60 (4.98) | 10.80 (5.36) | 9.40 (4.39) | 1.40 (1.67) |
| Super Bowl | 125 | 62 | 57 | 4 | 25.00 (10.82) | 12.20 (5.59) | 11.40 (6.54) | 0.10 (0.44) |
| Moderate Incidence period | | | | | | | | |
| Spring (Uni.)  Spring (Public)  Autumn (Uni.) | 275  141  59 | 83  27  0 | 45  9  0 | 38  18  0 | 19.64 (10.23)  14.10 (6.71)  1.20 (1.23) | 5.93 (6.15)  2.70 (3.34)  - | 3.21 (3.60)  0.90 (1.10)  - | 2.17 (3.17)  1.80 (2.57)  - |
| Easter | 70 | 12 | 3 | 9 | 14.00 (6.20) | 2.40 (3.05) | 0.60 (0.89) | 1.80 (2.49) |
| Passover | 127 | 9 | 3 | 6 | 12.70 (4.35) | 0.90 (1.29) | 0.30 (0.67) | 0.60 (1.26) |
| Sukkot | 28 | 0 | 0 | 0 | 5.60 (4.22) | - | - | - |
| Prophet’s Birthday | 83 | 29 | 14 | 15 | 16.60 (8.38) | 5.80 (6.02) | 2.80 (2.95) | 3.00 (3.67) |
| Ramadan | 138 | 0 | 0 | 0 | 5.31 (4.76) | - | - | - |
| Lailat al-Qadr | 31 | 0 | 0 | 0 | 6.20 (7.05) | - | - | - |
| Columbus Day | 22 | 0 | 0 | 0 | 4.40 (1.67) | - | - | - |
| Veteran’s Day | 43 | 0 | 0 | 0 | 8.60 (5.77) | - | - | - |
| Thanksgiving | 29 | 0 | 0 | 0 | 5.80 (2.77) | - | - | - |
| World Series | 57 | 0 | 0 | 0 | 5.70 (3.16) | - | - | - |
| Low Incidence period | | | | | | | | |
| Summer (Uni.) | 246 | 1 | 0 | 1 | 3.57 (2.73) | 0.01 (0.12) | - | 0.01 (0.12) |
| Summer (Public) | 210 | 1 | 0 | 1 | 3.33 (2.48) | 0.02 (013) | - | 0.02 (013) |
| Rosh Hashanah | 28 | 0 | 0 | 0 | 5.60 (3.03) | - | - | - |
| Yom Kippur | 22 | 0 | 0 | 0 | 4.40 (5.03) | - | - | - |
| Isra and Mi’raj | 22 | 0 | 0 | 0 | 4.40 (2.88) | - | - | - |
| Memorial Day | 17 | 0 | 0 | 0 | 3.40 (3.21) | - | - | - |
| Independence Day | 10 | 1 | 0 | 1 | 2.00 (1.00) | 0.20 (0.45) | - | 0.20 (0.45) |
| Labor Day | 15 | 0 | 0 | 0 | 3.00 (2.55) | - | - | - |
| Triple Crown | 104 | 0 | 0 | 0 | 8.00 (7.23) | - | - | - |
| NHL Finals | 46 | 0 | 0 | 0 | 4.18 (3.74) | - | - | - |
| NBA Finals | 59 | 0 | 0 | 0 | 4.54 (3.50) | - | - | - |

* Since holiday occurrences overlap, their sum does not add to the totals for the full study period.

** University calendar is abbreviated “Uni” while public-school calendar is abbreviated “Public”

Supplemental Table S4. Results of negative binomial regression modeling expressed as RRs (with 95%CI) for combined school holidays (marked as School) and individual school breaks (marked as Winter, Spring, Summer and Autumn) following the university (A) and public school system (B) calendars. The models were applied weekly tests in Milwaukee, WI (2004-2009).

| A | Model 1 | | | | | Model 2 | | | | | Model 3 | | | | |
| --- | --- | --- | --- | --- | --- | --- | --- | --- | --- | --- | --- | --- | --- | --- | --- |
|  | RR* | LCI | UCI | P-value | R^2^ | RR | LCI | UCI | P-value | R^2^ | RR | LCI | UCI | P-value | R^2^ |
| All Ages | | | | | | | | | | | | | | | |
| School | 0.49 | 0.39 | 0.63 | <0.005 | 0.02 | 0.72 | 0.57 | 0.91 | 0.01 | 0.07 | 0.72 | 0.59 | 0.88 | <0.005 | 0.12 |
| Winter | 0.40 | 0.30 | 0.53 | <0.005 | 0.06 | 0.33 | 0.23 | 0.45 | <0.005 | 0.10 | 0.31 | 0.23 | 0.40 | <0.005 | 0.15 |
| Spring | 1.64 | 1.22 | 2.21 | <0.005 |  | 1.46 | 1.12 | 1.91 | 0.01 |  | 1.10 | 0.78 | 1.53 | 0.59 |  |
| Summer | 0.30 | 0.24 | 0.37 | <0.005 |  | 0.71 | 0.52 | 0.95 | 0.02 |  | 0.92 | 0.68 | 1.26 | 0.62 |  |
| Autumn | 0.49 | 0.35 | 0.69 | <0.005 |  | 0.72 | 0.50 | 1.04 | 0.08 |  | 1.06 | 0.79 | 1.41 | 0.71 |  |
| ≤4 | | | | | | | | | | | | | | | |
| School | 0.79 | 0.62 | 1.02 | 0.07 | 0.00 | 0.86 | 0.66 | 1.13 | 0.29 | 0.00 | 0.90 | 0.68 | 1.18 | 0.45 | 0.03 |
| Winter | 0.67 | 0.42 | 1.07 | 0.09 | 0.01 | 0.62 | 0.38 | 0.99 | 0.04 | 0.01 | 0.73 | 0.43 | 1.24 | 0.24 | 0.03 |
| Spring | 1.14 | 0.69 | 1.88 | 0.61 |  | 1.07 | 0.66 | 1.75 | 0.78 |  | 0.85 | 0.49 | 1.48 | 0.57 |  |
| Summer | 0.80 | 0.59 | 1.08 | 0.15 |  | 1.06 | 0.70 | 1.60 | 0.79 |  | 1.08 | 0.69 | 1.69 | 0.73 |  |
| Autumn | 0.53 | 0.28 | 0.99 | 0.05 |  | 0.59 | 0.31 | 1.11 | 0.10 |  | 0.88 | 0.44 | 1.75 | 0.71 |  |
| 5-24 | | | | | | | | | | | | | | | |
| School | 0.33 | 0.23 | 0.47 | <0.005 | 0.04 | 0.54 | 0.39 | 0.75 | <0.005 | 0.10 | 0.53 | 0.40 | 0.71 | <0.005 | 0.14 |
| Winter | 0.22 | 0.14 | 0.35 | <0.005 | 0.09 | 0.16 | 0.10 | 0.26 | <0.005 | 0.13 | 0.14 | 0.09 | 0.23 | <0.005 | 0.17 |
| Spring | 1.38 | 0.89 | 2.14 | 0.15 |  | 1.24 | 0.84 | 1.84 | 0.27 |  | 0.90 | 0.59 | 1.38 | 0.64 |  |
| Summer | 0.15 | 0.11 | 0.20 | <0.005 |  | 0.52 | 0.36 | 0.75 | <0.005 |  | 0.79 | 0.52 | 1.19 | 0.26 |  |
| Autumn | 0.40 | 0.24 | 0.69 | <0.005 |  | 0.69 | 0.40 | 1.19 | 0.18 |  | 0.96 | 0.58 | 1.61 | 0.89 |  |
| 25-44 | | | | | | | | | | | | | | | |
| School | 0.47 | 0.32 | 0.70 | <0.005 | 0.02 | 0.65 | 0.43 | 0.98 | 0.04 | 0.06 | 0.65 | 0.45 | 0.94 | 0.02 | 0.11 |
| Winter | 0.38 | 0.21 | 0.69 | <0.005 | 0.08 | 0.31 | 0.16 | 0.60 | <0.005 | 0.09 | 0.31 | 0.17 | 0.56 | <0.005 | 0.13 |
| Spring | 1.92 | 1.26 | 2.94 | <0.005 |  | 1.75 | 1.14 | 2.69 | 0.01 |  | 1.21 | 0.72 | 2.03 | 0.47 |  |
| Summer | 0.19 | 0.11 | 0.31 | <0.005 |  | 0.36 | 0.18 | 0.70 | <0.005 |  | 0.47 | 0.22 | 0.98 | 0.04 |  |
| Autumn | 0.67 | 0.35 | 1.30 | 0.24 |  | 0.88 | 0.44 | 1.78 | 0.72 |  | 1.45 | 0.78 | 2.69 | 0.24 |  |
| 45-64 | | | | | | | | | | | | | | | |
| School | 0.84 | 0.51 | 1.38 | 0.49 | 0.00 | 1.18 | 0.71 | 1.96 | 0.52 | 0.04 | 1.12 | 0.73 | 1.74 | 0.60 | 0.08 |
| Winter | 0.67 | 0.40 | 1.13 | 0.13 | 0.05 | 0.54 | 0.31 | 0.94 | 0.03 | 0.07 | 0.51 | 0.28 | 0.95 | 0.03 | 0.1 |
| Spring | 3.46 | 1.65 | 7.25 | <0.005 |  | 3.06 | 1.46 | 6.40 | <0.005 |  | 2.05 | 0.94 | 4.47 | 0.07 |  |
| Summer | 0.37 | 0.19 | 0.69 | <0.005 |  | 0.77 | 0.35 | 1.66 | 0.50 |  | 1.03 | 0.45 | 2.37 | 0.94 |  |
| Autumn | 0.84 | 0.41 | 1.73 | 0.64 |  | 1.20 | 0.54 | 2.70 | 0.65 |  | 1.89 | 0.82 | 4.32 | 0.13 |  |
| ≥ 65 | | | | | | | | | | | | | | | |
| School | 0.57 | 0.31 | 1.05 | 0.07 | 0.01 | 0.74 | 0.42 | 1.33 | 0.31 | 0.04 | 0.79 | 0.46 | 1.36 | 0.39 | 0.09 |
| Winter | 0.75 | 0.29 | 1.93 | 0.55 | 0.05 | 0.64 | 0.23 | 1.83 | 0.41 | 0.05 | 0.56 | 0.23 | 1.34 | 0.19 | 0.1 |
| Spring | 2.33 | 1.13 | 4.83 | 0.02 |  | 2.06 | 0.97 | 4.37 | 0.06 |  | 2.13 | 0.86 | 5.26 | 0.10 |  |
| Summer | 0.19 | 0.08 | 0.43 | <0.005 |  | 0.33 | 0.13 | 0.83 | 0.02 |  | 0.39 | 0.15 | 0.98 | 0.05 |  |
| Autumn | 0.35 | 0.09 | 1.40 | 0.14 |  | 0.43 | 0.11 | 1.74 | 0.24 |  | 0.66 | 0.19 | 2.31 | 0.52 |  |

* Values shown in blue and red signify dampening (RR<1) and amplified (RR>1) effects.

| B | Model 1 | | | | | Model 2 | | | | | Model 3 | | | | |
| --- | --- | --- | --- | --- | --- | --- | --- | --- | --- | --- | --- | --- | --- | --- | --- |
|  | RR* | LCI | UCI | P-value | R^2^ | RR | LCI | UCI | P-value | R^2^ | RR | LCI | UCI | P-value | R^2^ |
| All Ages | | | | | | | | | | | | | | | |
| School | 0.45 | 0.36 | 0.58 | <0.005 | 0.02 | 0.72 | 0.57 | 0.91 | 0.01 | 0.07 | 0.69 | 0.55 | 0.85 | <0.005 | 0.12 |
| Winter | 0.38 | 0.26 | 0.56 | <0.005 | 0.05 | 0.31 | 0.19 | 0.49 | <0.005 | 0.08 | 0.28 | 0.20 | 0.40 | <0.005 | 0.14 |
| Spring | 1.22 | 0.89 | 1.66 | 0.21 |  | 1.19 | 0.88 | 1.62 | 0.26 |  | 0.98 | 0.69 | 1.38 | 0.90 |  |
| Summer | 0.29 | 0.23 | 0.36 | <0.005 |  | 0.69 | 0.52 | 0.92 | 0.01 |  | 0.92 | 0.68 | 1.25 | 0.59 |  |
| ≤4 | | | | | | | | | | | | | | | |
| School | 0.82 | 0.63 | 1.08 | 0.16 | 0.00 | 0.92 | 0.69 | 1.24 | 0.59 | 0.00 | 0.88 | 0.67 | 1.16 | 0.36 | 0.03 |
| Winter | 0.47 | 0.24 | 0.91 | 0.02 | 0.01 | 0.42 | 0.22 | 0.83 | 0.01 | 0.01 | 0.48 | 0.24 | 0.95 | 0.04 | 0.03 |
| Spring | 0.80 | 0.45 | 1.41 | 0.43 |  | 0.77 | 0.43 | 1.38 | 0.38 |  | 0.58 | 0.34 | 0.98 | 0.04 |  |
| Summer | 0.77 | 0.56 | 1.07 | 0.11 |  | 1.02 | 0.68 | 1.53 | 0.91 |  | 1.12 | 0.73 | 1.73 | 0.59 |  |
| 5-24 | | | | | | | | | | | | | | | |
| School | 0.26 | 0.19 | 0.36 | <0.005 | 0.05 | 0.49 | 0.37 | 0.65 | <0.005 | 0.10 | 0.48 | 0.36 | 0.63 | <0.005 | 0.14 |
| Winter | 0.21 | 0.12 | 0.36 | <0.005 | 0.07 | 0.15 | 0.08 | 0.28 | <0.005 | 0.12 | 0.14 | 0.08 | 0.23 | <0.005 | 0.16 |
| Spring | 0.95 | 0.66 | 1.35 | 0.76 |  | 1.01 | 0.65 | 1.58 | 0.95 |  | 0.90 | 0.57 | 1.43 | 0.66 |  |
| Summer | 0.15 | 0.11 | 0.20 | <0.005 |  | 0.53 | 0.37 | 0.77 | <0.005 |  | 0.81 | 0.54 | 1.21 | 0.30 |  |
| 25-44 | | | | | | | | | | | | | | | |
| School | 0.39 | 0.25 | 0.59 | <0.005 | 0.03 | 0.56 | 0.36 | 0.87 | 0.01 | 0.06 | 0.56 | 0.37 | 0.85 | 0.01 | 0.11 |
| Winter | 0.48 | 0.24 | 0.95 | 0.04 | 0.06 | 0.38 | 0.20 | 0.74 | <0.005 | 0.08 | 0.38 | 0.20 | 0.74 | <0.005 | 0.12 |
| Spring | 1.68 | 0.89 | 3.17 | 0.11 |  | 1.28 | 0.64 | 2.55 | 0.48 |  | 1.28 | 0.64 | 2.55 | 0.48 |  |
| Summer | 0.17 | 0.10 | 0.29 | <0.005 |  | 0.32 | 0.16 | 0.63 | <0.005 |  | 0.45 | 0.22 | 0.93 | 0.03 |  |
| 45-64 | | | | | | | | | | | | | | | |
| School | 0.71 | 0.41 | 1.25 | 0.24 | 0.00 | 1.03 | 0.58 | 1.82 | 0.92 | 0.04 | 0.95 | 0.57 | 1.57 | 0.83 | 0.08 |
| Winter | 0.48 | 0.23 | 1.01 | 0.05 | 0.05 | 0.40 | 0.18 | 0.88 | 0.02 | 0.07 | 0.38 | 0.18 | 0.80 | 0.01 | 0.09 |
| Spring | 2.70 | 1.06 | 6.91 | 0.04 |  | 2.47 | 0.99 | 6.17 | 0.05 |  | 1.89 | 0.75 | 4.77 | 0.17 |  |
| Summer | 0.25 | 0.14 | 0.43 | <0.005 |  | 0.46 | 0.22 | 0.95 | 0.04 |  | 0.65 | 0.30 | 1.43 | 0.28 |  |
| ≥ 65 | | | | | | | | | | | | | | | |
| School | 0.71 | 0.37 | 1.36 | 0.30 | 0.00 | 1.08 | 0.58 | 2.00 | 0.82 | 0.04 | 1.05 | 0.59 | 1.86 | 0.86 | 0.08 |
| Winter | 1.06 | 0.39 | 2.93 | 0.90 | 0.03 | 0.89 | 0.29 | 2.73 | 0.84 | 0.04 | 0.68 | 0.27 | 1.70 | 0.40 | 0.09 |
| Spring | 1.70 | 0.60 | 4.81 | 0.31 |  | 1.53 | 0.57 | 4.13 | 0.40 |  | 1.54 | 0.48 | 4.99 | 0.47 |  |
| Summer | 0.21 | 0.09 | 0.48 | <0.005 |  | 0.46 | 0.18 | 1.18 | 0.11 |  | 0.60 | 0.23 | 1.55 | 0.29 |  |

* Values shown in blue and red signify dampening (RR<1) and amplified (RR>1) effects

Supplemental Table S5. Peak timing estimates (with 95% CI) for four influenza health outcomes (tests, positives, influenza A and B positives) across six age groups for Models 3, 4, and 5.

|  | Tests | Positives | Influenza A | Influenza B |
| --- | --- | --- | --- | --- |
| Model 3 | | | | |
| All Ages | 6.33 | 6.63 | 6.06 | 10.36 |
|  | (5.15-7.50) | (5.91-7.36) | (5.31-6.81) | (9.09-11.63) |
| ≤ 4 | 12.37 | 5.01 | 4.32 | 12.18 |
|  | (3.11-21.63) | (2.17-7.86) | (2.98-5.67) | (12.13-12.23) |
| 5-24 | 5.07 | 6.91 | 6.53 | 10.17 |
|  | (3.99-6.16) | (6.13-7.68) | (5.72-7.34) | (8.28-12.05) |
| 25-44 | 7.08 | 6.63 | 5.85 | 9.87 |
|  | (4.43-9.72) | (5.05-8.20) | (4.15-7.54) | (7.31-12.43) |
| 45-64 | 6.29 | 6.43 | 5.87 | 10.94 |
|  | (4.07-8.51) | (4.69-8.17) | (3.72-8.02) | (9.84-12.04) |
| ≥ 65 | 7.10 | 7.00 | 6.24 | 10.62 |
|  | (3.71-10.48) | (5.36-8.64) | (4.30-8.18) | (9.20-12.03) |
| Model 4 | | | | |
| All Ages | 7.55 | 7.23 | 6.56 | 10.04 |
|  | (6.57 - 8.54) | (6.73-7.73) | (6.03-7.09) | (9.25-10.84) |
| ≤ 4 | 12.55 | 5.90 | 5.29 | 12.72 |
|  | (9.24-15.87) | (4.21-7.58) | (3.95-6.62) | * |
| 5-24 | 6.00 | 7.33 | 6.83 | 9.52 |
|  | (4.93-7.08) | (6.80-7.86) | (6.31-7.35) | (8.39-10.66) |
| 25-44 | 7.84 | 7.27 | 6.40 | 10.95 |
|  | (6.05-9.63) | (6.24-8.31) | (5.29-7.51) | (8.31-13.59) |
| 45-64 | 8.19 | 7.11 | 5.68 | 10.29 |
|  | (6.08-10.31) | (5.64-8.57) | (4.07-7.30) | (9.47-11.10) |
| ≥ 65 | 7.78 | 7.41 | 6.30 | 10.68 |
|  | (5.65-9.91) | (6.37-8.45) | (5.11-7.48) | (9.47-11.89) |
| Model 5 | | | | |
| All Ages | 7.55 | 7.23 | 6.56 | 10.04 |
|  | (6.57 - 8.54) | (6.73-7.73) | (6.03-7.09) | (9.25-10.84) |
| ≤ 4 | 12.55 | 5.90 | 5.29 | 12.72 |
|  | (9.24-15.87) | (4.21-7.58) | (3.95-6.62) | * |
| 5-24 | 6.00 | 7.33 | 6.83 | 9.52 |
|  | (4.93-7.08) | (6.80-7.86) | (6.31-7.35) | (8.39-10.66) |
| 25-44 | 7.84 | 7.27 | 6.40 | 10.95 |
|  | (6.05-9.63) | (6.24-8.31) | (5.29-7.51) | (8.31-13.59) |
| 45-64 | 8.19 | 7.11 | 5.68 | 10.29 |
|  | (6.08-10.31) | (5.64-8.57) | (4.07-7.30) | (9.47-11.10) |
| ≥ 65 | 7.78 | 7.41 | 6.30 | 10.68 |
|  | (5.65-9.91) | (6.37-8.45) | (5.11-7.48) | (9.47-11.89) |

* Only one influenza B positive case was detected for the ≤4 years age group

Supplemental Table S6. Average weekly counts across time periods related to university Winter and Spring school holiday breaks for the all ages and 5-24 years age groups.

| Period | All Ages | | | 5-24 Years | | | Period comparison |
| --- | --- | --- | --- | --- | --- | --- | --- |
|  | Mean | SD | P-value | Mean | SD | P-value |  |
| Tests | | | | | | | |
| Period 1 | 7.84 | 3.78 | - | 3.60 | 2.42 | - |  |
| Period 2 | 4.93 | 2.81 | 0.14 | 1.49 | 1.53 | 0.01 | 1 vs. 2 |
| Period 3 | 22.12 | 11.60 | <0.005 | 14.23 | 8.66 | <0.005 | 2 vs. 3 |
| Period 4 | 19.33 | 8.92 | 0.54 | 8.83 | 6.37 | 0.08 | 3 vs. 4 |
| Period 5 | 13.16 | 4.42 | 0.10 | 5.60 | 2.66 | 0.12 | 4 vs. 5 |
| Positives | | | | | | | |
| Period 1 | 0.04 | 0.20 | - | 0.00 | 0.00 | - |  |
| Period 2 | 0.67 | 0.82 | 0.06 | 0.29 | 0.47 | 0.03 | 1 vs. 2 |
| Period 3 | 9.61 | 7.16 | <0.005 | 6.60 | 5.43 | <0.005 | 2 vs. 3 |
| Period 4 | 5.93 | 5.02 | 0.12 | 3.67 | 4.55 | 0.11 | 3 vs. 4 |
| Period 5 | 1.336 | 1.88 | 0.06 | 0.72 | 1.03 | 0.15 | 4 vs. 5 |
| Influenza A | | | | | | | |
| Period 1 | 0.04 | 0.20 | - | 0.00 | 0.00 | - |  |
| Period 2 | 0.67 | 0.82 | 0.06 | 0.29 | 0.47 | 0.03 | 1 vs. 2 |
| Period 3 | 8.58 | 6.77 | <0.005 | 5.94 | 5.13 | <0.005 | 2 vs. 3 |
| Period 4 | 3.37 | 3.05 | 0.02 | 2.23 | 2.39 | 0.01 | 3 vs. 4 |
| Period 5 | 0.60 | 1.08 | 0.06 | 0.32 | 0.82 | 0.12 | 4 vs. 5 |
| Influenza B | | | | | | | |
| Period 1 | 0.00 | - | - | 0.00 | 0.00 | - |  |
| Period 2 | 0.00 | - | - | 0.00 | 0.00 | - | 1 vs. 2 |
| Period 3 | 1.03 | 1.19 | 0.08 | 0.67 | 0.67 | 0.12 | 2 vs. 3 |
| Period 4 | 2.57 | 2.48 | 0.11 | 1.43 | 2.33 | 0.32 | 3 vs. 4 |
| Period 5 | 0.76 | 1.25 | 0.10 | 0.40 | 0.75 | 0.25 | 4 vs. 5 |

Supplemental Table S7. Weekly counts and averages for the Christian (A), Jewish (B), and Muslim (C) observances categories (as well as their comparison periods without each category’s observances) for influenza tests, positives, A and B for six age groups in Milwaukee, WI (2004-2009).

| A | All Study Period | | | Weeks Without Christian Holidays | | | Weeks With Christian Holidays | | | MW Test | Percent Change* |
| --- | --- | --- | --- | --- | --- | --- | --- | --- | --- | --- | --- |
|  | (258 Weeks) | | | (242 Weeks) | | | (16 Weeks) | | |  |  |
|  | Counts | Mean | SD | Counts | Mean | SD | Counts | Mean | SD | P-Value | % |
| Tests | | | | | | | | | | | |
| All Ages | 2378 | 9.22 | 9.24 | 2139 | 8.84 | 8.90 | 239 | 14.94 | 12.47 | 0.08 | 69 |
| ≤ 4 | 527 | 2.04 | 2.07 | 509 | 2.10 | 2.08 | 18 | 1.13 | 1.75 | 0.05 | -47 |
| 5-24 | 1153 | 4.47 | 6.12 | 1001 | 4.14 | 5.57 | 152 | 9.50 | 10.69 | 0.16 | 130 |
| 25-44 | 291 | 1.13 | 1.70 | 259 | 1.07 | 1.68 | 32 | 2.00 | 1.83 | 0.01 | 87 |
| 45-64 | 227 | 0.88 | 1.66 | 210 | 0.87 | 1.64 | 17 | 1.06 | 2.05 | 0.98 | 22 |
| ≥ 65 | 178 | 0.69 | 1.74 | 158 | 0.65 | 1.70 | 20 | 1.25 | 2.32 | 0.21 | 91 |
| Positive | | | | | | | | | | | |
| All Ages | 505 | 1.96 | 4.68 | 419 | 1.73 | 4.40 | 86 | 5.38 | 7.21 | <0.005 | 210 |
| ≤ 4 | 12 | 0.05 | 0.23 | 11 | 0.05 | 0.23 | 1 | 0.06 | 0.25 | 0.69 | 38 |
| 5-24 | 326 | 1.26 | 3.39 | 267 | 1.10 | 3.12 | 59 | 3.69 | 5.85 | 0.03 | 234 |
| 25-44 | 87 | 0.34 | 0.92 | 75 | 0.31 | 0.92 | 12 | 0.75 | 0.86 | <0.005 | 142 |
| 45-64 | 32 | 0.12 | 0.56 | 26 | 0.11 | 0.53 | 6 | 0.38 | 0.89 | 0.06 | 249 |
| ≥ 65 | 47 | 0.18 | 1.03 | 39 | 0.16 | 1.03 | 8 | 0.50 | 1.10 | 0.01 | 210 |
| Influenza A | | | | | | | | | | | |
| All Ages | 410 | 1.59 | 4.10 | 338 | 1.40 | 3.80 | 72 | 4.50 | 6.80 | <0.005 | 222 |
| ≤ 4 | 11 | 0.04 | 0.22 | 10 | 0.04 | 0.22 | 1 | 0.06 | 0.25 | 0.62 | 51 |
| 5-24 | 272 | 1.05 | 2.93 | 220 | 0.91 | 2.65 | 52 | 3.25 | 5.41 | 0.06 | 258 |
| 25-44 | 67 | 0.26 | 0.81 | 55 | 0.23 | 0.80 | 12 | 0.75 | 0.86 | <0.005 | 230 |
| 45-64 | 21 | 0.08 | 0.38 | 19 | 0.08 | 0.37 | 2 | 0.13 | 0.50 | 0.90 | 59 |
| ≥ 65 | 38 | 0.15 | 0.97 | 33 | 0.14 | 0.97 | 5 | 0.31 | 1.01 | 0.27 | 129 |
| Influenza B | | | | | | | | | | | |
| All Ages | 95 | 0.37 | 1.20 | 81 | 0.33 | 1.15 | 14 | 0.88 | 1.75 | 0.12 | 161 |
| ≤ 4 | 1 | 0.00 | 0.06 | 1 | 0.00 | 0.06 | 0 | 0.00 | 0.00 | 0.80 | -100 |
| 5-24 | 54 | 0.21 | 0.85 | 47 | 0.19 | 0.83 | 7 | 0.44 | 1.03 | 0.21 | 125 |
| 25-44 | 20 | 0.08 | 0.38 | 20 | 0.08 | 0.39 | 0 | 0.00 | 0.00 | 0.36 | -100 |
| 45-64 | 11 | 0.04 | 0.33 | 7 | 0.03 | 0.28 | 4 | 0.25 | 0.77 | 0.01 | 764 |
| ≥ 65 | 9 | 0.03 | 0.25 | 6 | 0.02 | 0.22 | 3 | 0.19 | 0.54 | 0.01 | 656 |

* Percent Change = ((Mean Cases Christian Holiday Weeks – Mean Cases No Christian Holidays Weeks) / Mean Cases No Christian Holidays Weeks)*100%

| B | All Study Period | | | Weeks Without Jewish Holidays | | | Weeks With Jewish Holidays | | | MW Test | Percent Change* |
| --- | --- | --- | --- | --- | --- | --- | --- | --- | --- | --- | --- |
|  | (258 Weeks) | | | (223 Weeks) | | | (35 Weeks) | | |  |  |
|  | Counts | Mean | SD | Counts | Mean | SD | Counts | Mean | SD | P-Value | % |
| Tests | | | | | | | | | | | |
| All Ages | 2378 | 9.22 | 9.24 | 2114 | 9.48 | 9.69 | 264 | 7.54 | 5.44 | 0.78 | -20 |
| ≤ 4 | 527 | 2.04 | 2.07 | 468 | 2.10 | 2.11 | 59 | 1.69 | 1.76 | 0.38 | -20 |
| 5-24 | 1153 | 4.47 | 6.12 | 1035 | 4.64 | 6.46 | 118 | 3.37 | 3.05 | 0.66 | -27 |
| 25-44 | 291 | 1.13 | 1.70 | 256 | 1.15 | 1.75 | 35 | 1.00 | 1.39 | 0.79 | -13 |
| 45-64 | 227 | 0.88 | 1.66 | 203 | 0.91 | 1.74 | 24 | 0.69 | 1.08 | 0.50 | -25 |
| ≥ 65 | 178 | 0.69 | 1.74 | 150 | 0.67 | 1.80 | 28 | 0.80 | 1.32 | 0.17 | 19 |
| Positive | | | | | | | | | | | |
| All Ages | 505 | 1.96 | 4.68 | 490 | 2.20 | 4.98 | 15 | 0.43 | 0.95 | 0.19 | -81 |
| ≤ 4 | 12 | 0.05 | 0.23 | 12 | 0.05 | 0.25 | 0 | 0.00 | 0.00 | 0.18 | -100 |
| 5-24 | 326 | 1.26 | 3.39 | 319 | 1.43 | 3.61 | 7 | 0.20 | 0.58 | 0.08 | -86 |
| 25-44 | 87 | 0.34 | 0.92 | 83 | 0.37 | 0.98 | 4 | 0.11 | 0.40 | 0.16 | -69 |
| 45-64 | 32 | 0.12 | 0.56 | 30 | 0.13 | 0.59 | 2 | 0.06 | 0.24 | 0.66 | -58 |
| ≥ 65 | 47 | 0.18 | 1.03 | 45 | 0.20 | 1.11 | 2 | 0.06 | 0.24 | 0.55 | -72 |
| Influenza A | | | | | | | | | | | |
| All Ages | 410 | 1.59 | 4.10 | 401 | 1.80 | 4.37 | 9 | 0.26 | 0.70 | 0.06 | -86 |
| ≤ 4 | 11 | 0.04 | 0.22 | 11 | 0.05 | 0.24 | 0 | 0.00 | 0.00 | 0.20 | -100 |
| 5-24 | 272 | 1.05 | 2.93 | 270 | 1.21 | 3.12 | 2 | 0.06 | 0.24 | 0.01 | -95 |
| 25-44 | 67 | 0.26 | 0.81 | 63 | 0.28 | 0.85 | 4 | 0.11 | 0.40 | 0.31 | -60 |
| 45-64 | 21 | 0.08 | 0.38 | 19 | 0.09 | 0.40 | 2 | 0.06 | 0.24 | 0.96 | -33 |
| ≥ 65 | 38 | 0.15 | 0.97 | 37 | 0.17 | 1.04 | 1 | 0.03 | 0.17 | 0.37 | -83 |
| Influenza B | | | | | | | | | | | |
| All Ages | 95 | 0.37 | 1.20 | 89 | 0.40 | 1.25 | 6 | 0.17 | 0.71 | 0.36 | -57 |
| ≤ 4 | 1 | 0.00 | 0.06 | 1 | 0.00 | 0.07 | 0 | 0.00 | 0.00 | 0.69 | -100 |
| 5-24 | 54 | 0.21 | 0.85 | 49 | 0.22 | 0.89 | 5 | 0.14 | 0.55 | 0.74 | -35 |
| 25-44 | 20 | 0.08 | 0.38 | 20 | 0.09 | 0.40 | 0 | 0.00 | 0.00 | 0.16 | -100 |
| 45-64 | 11 | 0.04 | 0.33 | 11 | 0.05 | 0.36 | 0 | 0.00 | 0.00 | 0.33 | -100 |
| ≥ 65 | 9 | 0.03 | 0.25 | 8 | 0.04 | 0.27 | 1 | 0.03 | 0.17 | 0.83 | -20 |

* Percent Change = ((Mean Cases Jewish Holiday Weeks – Mean Cases No Jewish Holidays Weeks) / Mean Cases No Jewish Holidays Weeks)*100%

| C | All Study Period | | | Weeks Without Muslim Holidays | | | Weeks With Muslim Holidays | | | MW Test | Percent Change* |
| --- | --- | --- | --- | --- | --- | --- | --- | --- | --- | --- | --- |
|  | (258 Weeks) | | | (212 Weeks) | | | (46 Weeks) | | |  |  |
|  | Counts | Mean | SD | Counts | Mean | SD | Counts | Mean | SD | P-Value | % |
| Tests | | | | | | | | | | | |
| All Ages | 2378 | 9.22 | 9.24 | 2042 | 9.63 | 9.52 | 336 | 7.30 | 7.64 | 0.10 | -24 |
| ≤ 4 | 527 | 2.04 | 2.07 | 457 | 2.16 | 2.12 | 70 | 1.52 | 1.79 | 0.06 | -29 |
| 5-24 | 1153 | 4.47 | 6.12 | 988 | 4.66 | 6.37 | 165 | 3.59 | 4.78 | 0.86 | -23 |
| 25-44 | 291 | 1.13 | 1.70 | 255 | 1.20 | 1.76 | 36 | 0.78 | 1.33 | 0.12 | -35 |
| 45-64 | 227 | 0.88 | 1.66 | 187 | 0.88 | 1.58 | 40 | 0.87 | 2.02 | 0.63 | -1 |
| ≥ 65 | 178 | 0.69 | 1.74 | 153 | 0.72 | 1.83 | 25 | 0.54 | 1.26 | 0.70 | -25 |
| Positive | | | | | | | | | | | |
| All Ages | 505 | 1.96 | 4.68 | 447 | 2.11 | 4.87 | 58 | 1.26 | 3.67 | 0.18 | -40 |
| ≤ 4 | 12 | 0.05 | 0.23 | 11 | 0.05 | 0.24 | 1 | 0.02 | 0.15 | 0.44 | -58 |
| 5-24 | 326 | 1.26 | 3.39 | 292 | 1.38 | 3.55 | 34 | 0.74 | 2.50 | 0.47 | -46 |
| 25-44 | 87 | 0.34 | 0.92 | 80 | 0.38 | 0.99 | 7 | 0.15 | 0.51 | 0.23 | -60 |
| 45-64 | 32 | 0.12 | 0.56 | 23 | 0.11 | 0.49 | 9 | 0.20 | 0.81 | 0.68 | 80 |
| ≥ 65 | 47 | 0.18 | 1.03 | 40 | 0.19 | 1.10 | 7 | 0.15 | 0.67 | 0.69 | -19 |
| Influenza A | | | | | | | | | | | |
| All Ages | 410 | 1.59 | 4.10 | 367 | 1.73 | 4.30 | 43 | 0.93 | 2.98 | 0.26 | -46 |
| ≤ 4 | 11 | 0.04 | 0.22 | 10 | 0.05 | 0.23 | 1 | 0.02 | 0.15 | 0.51 | -54 |
| 5-24 | 272 | 1.05 | 2.93 | 245 | 1.16 | 3.10 | 27 | 0.59 | 1.94 | 0.55 | -49 |
| 25-44 | 67 | 0.26 | 0.81 | 63 | 0.30 | 0.88 | 4 | 0.09 | 0.28 | 0.22 | -71 |
| 45-64 | 21 | 0.08 | 0.38 | 16 | 0.08 | 0.38 | 5 | 0.11 | 0.38 | 0.36 | 44 |
| ≥ 65 | 38 | 0.15 | 0.97 | 32 | 0.15 | 1.03 | 6 | 0.13 | 0.65 | 0.60 | -14 |
| Influenza B | | | | | | | | | | | |
| All Ages | 95 | 0.37 | 1.20 | 80 | 0.38 | 1.14 | 15 | 0.33 | 1.45 | 0.16 | -14 |
| ≤ 4 | 1 | 0.00 | 0.06 | 1 | 0.00 | 0.07 | 0 | 0.00 | 0.00 | 0.64 | -100 |
| 5-24 | 54 | 0.21 | 0.85 | 47 | 0.22 | 0.84 | 7 | 0.15 | 0.89 | 0.16 | -31 |
| 25-44 | 20 | 0.08 | 0.38 | 17 | 0.08 | 0.39 | 3 | 0.07 | 0.33 | 0.91 | -19 |
| 45-64 | 11 | 0.04 | 0.33 | 7 | 0.03 | 0.25 | 4 | 0.09 | 0.59 | 0.96 | 163 |
| ≥ 65 | 9 | 0.03 | 0.25 | 8 | 0.04 | 0.27 | 1 | 0.02 | 0.15 | 0.93 | -42 |

* Percent Change = ((Mean Cases Muslim Holiday Weeks – Mean Cases No Muslim Holidays Weeks) / Mean Cases No Muslim Holidays Weeks)*100%

Supplemental Table S8. Weekly counts and averages for the Federal (A) and Sporting Event (B) observances categories (as well as their comparison periods without each category’s observances) for influenza tests, positives, A and B for six age groups in Milwaukee, WI (2004-2009).

| A | All Study Period | | | Weeks Without Federal Holidays | | | Weeks With Federal Holidays | | | MW Test | Percent Change* |
| --- | --- | --- | --- | --- | --- | --- | --- | --- | --- | --- | --- |
|  | (258 Weeks) | | | (213 Weeks) | | | (45 Weeks) | | |  |  |
|  | Counts | Mean | SD | Counts | Mean | SD | Counts | Mean | SD | P-Value | % |
| Tests | | | | | | | | | | | |
| All Ages | 2378 | 9.22 | 9.24 | 2020 | 9.48 | 9.49 | 358 | 7.96 | 7.93 | 0.25 | -16 |
| ≤ 4 | 527 | 2.04 | 2.07 | 458 | 2.15 | 2.07 | 69 | 1.53 | 2.02 | 0.04 | -29 |
| 5-24 | 1153 | 4.47 | 6.12 | 967 | 4.54 | 6.13 | 186 | 4.13 | 6.11 | 0.47 | -9 |
| 25-44 | 291 | 1.13 | 1.70 | 249 | 1.17 | 1.80 | 42 | 0.93 | 1.14 | 0.97 | -20 |
| 45-64 | 227 | 0.88 | 1.66 | 193 | 0.91 | 1.76 | 34 | 0.76 | 1.11 | 0.95 | -17 |
| ≥ 65 | 178 | 0.69 | 1.74 | 151 | 0.71 | 1.83 | 27 | 0.60 | 1.23 | 0.91 | -15 |
| Positive | | | | | | | | | | | |
| All Ages | 505 | 1.96 | 4.68 | 429 | 2.01 | 4.83 | 76 | 1.69 | 3.95 | 0.69 | -16 |
| ≤ 4 | 12 | 0.05 | 0.23 | 10 | 0.05 | 0.23 | 2 | 0.04 | 0.21 | 0.95 | -5 |
| 5-24 | 326 | 1.26 | 3.39 | 275 | 1.29 | 3.45 | 51 | 1.13 | 3.16 | 0.92 | -12 |
| 25-44 | 87 | 0.34 | 0.92 | 76 | 0.36 | 0.97 | 11 | 0.24 | 0.68 | 0.56 | -31 |
| 45-64 | 32 | 0.12 | 0.56 | 28 | 0.13 | 0.60 | 4 | 0.09 | 0.29 | 0.71 | -32 |
| ≥ 65 | 47 | 0.18 | 1.03 | 39 | 0.18 | 1.12 | 8 | 0.18 | 0.49 | 0.17 | -3 |
| Influenza A | | | | | | | | | | | |
| All Ages | 410 | 1.59 | 4.10 | 342 | 1.61 | 4.23 | 68 | 1.51 | 3.47 | 0.91 | -6 |
| ≤ 4 | 11 | 0.04 | 0.22 | 9 | 0.04 | 0.22 | 2 | 0.04 | 0.21 | 0.83 | 5 |
| 5-24 | 272 | 1.05 | 2.93 | 225 | 1.06 | 2.96 | 47 | 1.04 | 2.84 | 0.71 | -1 |
| 25-44 | 67 | 0.26 | 0.81 | 59 | 0.28 | 0.84 | 8 | 0.18 | 0.61 | 0.31 | -36 |
| 45-64 | 21 | 0.08 | 0.38 | 18 | 0.08 | 0.40 | 3 | 0.07 | 0.25 | 0.81 | -21 |
| ≥ 65 | 38 | 0.15 | 0.97 | 30 | 0.14 | 1.05 | 8 | 0.18 | 0.49 | 0.03 | 26 |
| Influenza B | | | | | | | | | | | |
| All Ages | 95 | 0.37 | 1.20 | 87 | 0.41 | 1.28 | 8 | 0.18 | 0.68 | 0.32 | -56 |
| ≤ 4 | 1 | 0.00 | 0.06 | 1 | 0.00 | 0.07 | 0 | 0.00 | 0.00 | 0.65 | -100 |
| 5-24 | 54 | 0.21 | 0.85 | 50 | 0.23 | 0.92 | 4 | 0.09 | 0.36 | 0.38 | -62 |
| 25-44 | 20 | 0.08 | 0.38 | 17 | 0.08 | 0.39 | 3 | 0.07 | 0.33 | 0.94 | -16 |
| 45-64 | 11 | 0.04 | 0.33 | 10 | 0.05 | 0.36 | 1 | 0.02 | 0.15 | 0.95 | -53 |
| ≥ 65 | 9 | 0.03 | 0.25 | 9 | 0.04 | 0.28 | 0 | 0.00 | 0.00 | 0.26 | -100 |

* Percent Change = ((Mean Cases Federal Holiday Weeks – Mean Cases No Federal Holidays Weeks) / Mean Cases No Federal Holidays Weeks)*100%

| B | All Study Period | | | Weeks Without Sporting Events | | | Weeks With Sporting Events | | | MW Test | Percent Change* |
| --- | --- | --- | --- | --- | --- | --- | --- | --- | --- | --- | --- |
|  | (258 Weeks) | | | (217 Weeks) | | | (41 Weeks) | | |  |  |
|  | Counts | Mean | SD | Counts | Mean | SD | Counts | Mean | SD | P-Value | % |
| Tests | | | | | | | | | | | |
| All Ages | 2378 | 9.22 | 9.24 | 2041 | 9.41 | 9.37 | 337 | 8.22 | 8.60 | 0.42 | -13% |
| ≤ 4 | 527 | 2.04 | 2.07 | 441 | 2.03 | 2.11 | 86 | 2.10 | 1.89 | 0.62 | 3% |
| 5-24 | 1153 | 4.47 | 6.12 | 995 | 4.59 | 6.04 | 158 | 3.85 | 6.56 | 0.07 | -16% |
| 25-44 | 291 | 1.13 | 1.70 | 248 | 1.14 | 1.73 | 43 | 1.05 | 1.53 | 0.72 | -8% |
| 45-64 | 227 | 0.88 | 1.66 | 200 | 0.92 | 1.73 | 27 | 0.66 | 1.22 | 0.22 | -29% |
| ≥ 65 | 178 | 0.69 | 1.74 | 155 | 0.71 | 1.82 | 23 | 0.56 | 1.27 | 0.84 | -21% |
| Positive | | | | | | | | | | | |
| All Ages | 505 | 1.96 | 4.68 | 444 | 2.05 | 4.74 | 61 | 1.49 | 4.41 | 0.02 | -27% |
| ≤ 4 | 12 | 0.05 | 0.23 | 11 | 0.05 | 0.24 | 1 | 0.02 | 0.16 | 0.53 | -52% |
| 5-24 | 326 | 1.26 | 3.39 | 284 | 1.31 | 3.46 | 42 | 1.02 | 3.06 | 0.08 | -22% |
| 25-44 | 87 | 0.34 | 0.92 | 76 | 0.35 | 0.93 | 11 | 0.27 | 0.90 | 0.26 | -23% |
| 45-64 | 32 | 0.12 | 0.56 | 30 | 0.14 | 0.59 | 2 | 0.05 | 0.31 | 0.20 | -65% |
| ≥ 65 | 47 | 0.18 | 1.03 | 42 | 0.19 | 1.09 | 5 | 0.12 | 0.64 | 0.42 | -37% |
| Influenza A | | | | | | | | | | | |
| All Ages | 410 | 1.59 | 4.10 | 353 | 1.63 | 4.07 | 57 | 1.39 | 4.31 | 0.06 | -15% |
| ≤ 4 | 11 | 0.04 | 0.22 | 10 | 0.05 | 0.23 | 1 | 0.02 | 0.16 | 0.60 | -47% |
| 5-24 | 272 | 1.05 | 2.93 | 234 | 1.08 | 2.93 | 38 | 0.93 | 2.96 | 0.16 | -14% |
| 25-44 | 67 | 0.26 | 0.81 | 56 | 0.26 | 0.79 | 11 | 0.27 | 0.90 | 0.47 | 4% |
| 45-64 | 21 | 0.08 | 0.38 | 19 | 0.09 | 0.39 | 2 | 0.05 | 0.31 | 0.33 | -44% |
| ≥ 65 | 38 | 0.15 | 0.97 | 33 | 0.15 | 1.02 | 5 | 0.12 | 0.64 | 0.72 | -20% |
| Influenza B | | | | | | | | | | | |
| All Ages | 95 | 0.37 | 1.20 | 91 | 0.42 | 1.29 | 4 | 0.10 | 0.44 | 0.09 | -77% |
| ≤ 4 | 1 | 0.00 | 0.06 | 1 | 0.00 | 0.07 | 0 | 0.00 | 0.00 | 0.66 | -100% |
| 5-24 | 54 | 0.21 | 0.85 | 50 | 0.23 | 0.90 | 4 | 0.10 | 0.44 | 0.24 | -58% |
| 25-44 | 20 | 0.08 | 0.38 | 20 | 0.09 | 0.41 | 0 | 0.00 | 0.00 | 0.12 | -100% |
| 45-64 | 11 | 0.04 | 0.33 | 11 | 0.05 | 0.36 | 0 | 0.00 | 0.00 | 0.28 | -100% |
| ≥ 65 | 9 | 0.03 | 0.25 | 9 | 0.04 | 0.28 | 0 | 0.00 | 0.00 | 0.28 | -100% |

* Percent Change = ((Mean Cases Sporting Event Weeks – Mean Cases No Sporting Event Weeks) / Mean Cases No Sporting Event Weeks)*100%

Supplemental Table S9. Results of the negative binomial regression modeling, expressed as RRs with 95%CI for all holiday categories (i.e. School, Christian, Jewish, Muslim, Federal, and Sporting). The models were applied to weekly tests in Milwaukee, WI (2004-2009).

|  | Model 1 | | | | | Model 2 | | | | | Model 3 | | | | |
| --- | --- | --- | --- | --- | --- | --- | --- | --- | --- | --- | --- | --- | --- | --- | --- |
| Tests | RR | LCI | UCI | P-value | R^2^ | RR | LCI | UCI | P-value | R^2^ | RR | LCI | UCI | P-value | R^2^ |
| All Ages | | | | | | | | | | | | | | | |
| Christian | 1.69 | 1.11 | 2.56 | 0.01 | 0.00 | 1.19 | 0.79 | 1.81 | 0.40 | 0.06 | 0.94 | 0.64 | 1.38 | 0.75 | 0.11 |
| Jewish | 0.80 | 0.61 | 1.04 | 0.10 | 0.00 | 0.88 | 0.67 | 1.15 | 0.34 | 0.06 | 0.99 | 0.77 | 1.26 | 0.91 | 0.11 |
| Muslim | 0.76 | 0.55 | 1.05 | 0.10 | 0.00 | 0.87 | 0.67 | 1.12 | 0.28 | 0.07 | 0.99 | 0.78 | 1.26 | 0.95 | 0.11 |
| Federal | 0.84 | 0.61 | 1.15 | 0.28 | 0.00 | 0.71 | 0.57 | 0.89 | <0.005 | 0.07 | 0.84 | 0.67 | 1.03 | 0.10 | 0.11 |
| Sporting | 0.87 | 0.62 | 1.23 | 0.44 | 0.00 | 1.05 | 0.79 | 1.38 | 0.76 | 0.06 | 0.97 | 0.78 | 1.21 | 0.79 | 0.11 |
| ≤4 | | | | | | | | | | | | | | | |
| Christian | 0.53 | 0.25 | 1.13 | 0.10 | 0.00 | 0.47 | 0.22 | 0.98 | 0.05 | 0.01 | 0.43 | 0.20 | 0.91 | 0.03 | 0.03 |
| Jewish | 0.80 | 0.56 | 1.16 | 0.24 | 0.00 | 0.79 | 0.55 | 1.14 | 0.21 | 0.00 | 0.85 | 0.61 | 1.19 | 0.34 | 0.03 |
| Muslim | 0.71 | 0.49 | 1.01 | 0.06 | 0.00 | 0.72 | 0.50 | 1.04 | 0.08 | 0.01 | 0.86 | 0.60 | 1.24 | 0.42 | 0.03 |
| Federal | 0.71 | 0.48 | 1.07 | 0.10 | 0.00 | 0.67 | 0.46 | 0.97 | 0.03 | 0.01 | 0.77 | 0.52 | 1.14 | 0.19 | 0.03 |
| Sporting | 1.03 | 0.76 | 1.40 | 0.84 | 0.00 | 1.09 | 0.80 | 1.50 | 0.58 | 0.00 | 0.92 | 0.67 | 1.25 | 0.60 | 0.03 |
| 5-24 | | | | | | | | | | | | | | | |
| Christian | 2.30 | 1.31 | 4.03 | <0.005 | 0.01 | 1.54 | 0.89 | 2.67 | 0.12 | 0.09 | 1.16 | 0.69 | 1.94 | 0.58 | 0.12 |
| Jewish | 0.73 | 0.51 | 1.03 | 0.07 | 0.00 | 0.94 | 0.66 | 1.35 | 0.75 | 0.09 | 1.11 | 0.76 | 1.63 | 0.58 | 0.12 |
| Muslim | 0.77 | 0.50 | 1.18 | 0.23 | 0.00 | 1.06 | 0.76 | 1.46 | 0.74 | 0.09 | 1.15 | 0.82 | 1.61 | 0.41 | 0.12 |
| Federal | 0.91 | 0.57 | 1.45 | 0.69 | 0.00 | 0.77 | 0.55 | 1.08 | 0.13 | 0.09 | 0.87 | 0.63 | 1.20 | 0.40 | 0.12 |
| Sporting | 0.84 | 0.49 | 1.45 | 0.53 | 0.00 | 0.95 | 0.62 | 1.47 | 0.83 | 0.09 | 0.94 | 0.68 | 1.30 | 0.73 | 0.12 |
| 25-44 | | | | | | | | | | | | | | | |
| Christian | 1.87 | 1.16 | 3.01 | 0.01 | 0.00 | 1.36 | 0.76 | 2.44 | 0.31 | 0.05 | 1.02 | 0.63 | 1.65 | 0.94 | 0.10 |
| Jewish | 0.87 | 0.53 | 1.43 | 0.59 | 0.00 | 0.87 | 0.53 | 1.44 | 0.60 | 0.05 | 0.95 | 0.62 | 1.44 | 0.80 | 0.10 |
| Muslim | 0.65 | 0.38 | 1.10 | 0.11 | 0.00 | 0.74 | 0.46 | 1.22 | 0.24 | 0.06 | 0.84 | 0.49 | 1.45 | 0.54 | 0.10 |
| Federal | 0.80 | 0.53 | 1.20 | 0.28 | 0.00 | 0.72 | 0.47 | 1.10 | 0.13 | 0.06 | 0.87 | 0.58 | 1.32 | 0.52 | 0.10 |
| Sporting | 0.92 | 0.56 | 1.49 | 0.73 | 0.00 | 1.04 | 0.67 | 1.64 | 0.85 | 0.05 | 1.06 | 0.69 | 1.62 | 0.80 | 0.10 |
| 45-64 | | | | | | | | | | | | | | | |
| Christian | 1.22 | 0.48 | 3.16 | 0.68 | 0.00 | 0.84 | 0.32 | 2.20 | 0.72 | 0.04 | 0.65 | 0.26 | 1.64 | 0.36 | 0.08 |
| Jewish | 0.75 | 0.43 | 1.33 | 0.33 | 0.00 | 0.78 | 0.44 | 1.39 | 0.40 | 0.04 | 0.84 | 0.49 | 1.44 | 0.54 | 0.08 |
| Muslim | 0.99 | 0.49 | 2.00 | 0.97 | 0.00 | 1.02 | 0.55 | 1.92 | 0.94 | 0.04 | 1.19 | 0.65 | 2.18 | 0.57 | 0.08 |
| Federal | 0.83 | 0.51 | 1.37 | 0.48 | 0.00 | 0.75 | 0.45 | 1.23 | 0.26 | 0.04 | 0.98 | 0.60 | 1.57 | 0.92 | 0.08 |
| Sporting | 0.71 | 0.39 | 1.32 | 0.28 | 0.00 | 1.02 | 0.52 | 2.01 | 0.95 | 0.04 | 0.94 | 0.50 | 1.76 | 0.85 | 0.08 |
| ≥ 65 | | | | | | | | | | | | | | | |
| Christian | 1.91 | 0.75 | 4.91 | 0.18 | 0.00 | 1.25 | 0.47 | 3.34 | 0.66 | 0.04 | 1.02 | 0.39 | 2.67 | 0.97 | 0.08 |
| Jewish | 1.19 | 0.62 | 2.27 | 0.60 | 0.00 | 1.45 | 0.77 | 2.73 | 0.25 | 0.04 | 1.85 | 0.95 | 3.61 | 0.07 | 0.09 |
| Muslim | 0.75 | 0.36 | 1.59 | 0.46 | 0.00 | 0.76 | 0.39 | 1.48 | 0.41 | 0.04 | 0.87 | 0.45 | 1.69 | 0.69 | 0.08 |
| Federal | 0.85 | 0.43 | 1.68 | 0.63 | 0.00 | 0.67 | 0.33 | 1.35 | 0.26 | 0.04 | 0.78 | 0.41 | 1.49 | 0.45 | 0.08 |
| Sporting | 0.79 | 0.37 | 1.68 | 0.53 | 0.00 | 0.99 | 0.50 | 1.97 | 0.98 | 0.04 | 0.87 | 0.48 | 1.58 | 0.65 | 0.08 |

* Values shown in blue and red signify dampening (RR<1) and amplified (RR>1) effects.

Supplemental Table S10. Results of the negative binomial regression modeling, expressed as RRs with 95%CI for tests and positives across three Christian observances (Christmas, Ash Wednesday, and Easter) for three age groups (all ages, 5-24 years, and 25-44 years) for Milwaukee, WI (2004-2009).

|  | Model 1 | | | | | Model 2 | | | | | Model 3 | | | | |
| --- | --- | --- | --- | --- | --- | --- | --- | --- | --- | --- | --- | --- | --- | --- | --- |
|  | RR | LCI | UCI | P-value | R^2^ | RR | LCI | UCI | P-value | R^2^ | RR | LCI | UCI | P-value | R^2^ |
| Tests | | | | | | | | | | | | | | | |
| All Ages | | | | | | | | | | | | | | | |
| Christmas | 0.32 | 0.19 | 0.54 | <0.005 | 0.00 | 0.19 | 0.11 | 0.33 | <0.005 | 0.07 | 0.22 | 0.14 | 0.33 | <0.005 | 0.12 |
| Ash Wednesday | 3.43 | 2.83 | 4.17 | <0.005 | 0.01 | 2.28 | 1.74 | 2.99 | <0.005 | 0.07 | 1.75 | 1.34 | 2.30 | <0.005 | 0.11 |
| Easter | 1.53 | 1.06 | 2.22 | 0.02 | 0.00 | 1.36 | 0.92 | 2.00 | 0.12 | 0.06 | 1.05 | 0.68 | 1.60 | 0.84 | 0.11 |
| 5-24 | | | | | | | | | | | | | | | |
| Christmas | 0.11 | 0.02 | 0.66 | 0.02 | 0.01 | 0.04 | 0.01 | 0.16 | <0.005 | 0.11 | 0.06 | 0.01 | 0.30 | <0.005 | 0.14 |
| Ash Wednesday | 5.71 | 4.39 | 7.44 | <0.005 | 0.01 | 3.54 | 2.54 | 4.94 | <0.005 | 0.10 | 2.62 | 1.85 | 3.71 | <0.005 | 0.13 |
| Easter | 1.44 | 0.88 | 2.37 | 0.15 | 0.00 | 1.44 | 0.77 | 2.70 | 0.26 | 0.09 | 1.14 | 0.61 | 2.10 | 0.69 | 0.12 |
| 25-44 | | | | | | | | | | | | | | | |
| Christmas | 0.59 | 0.23 | 1.46 | 0.25 | 0.00 | 0.33 | 0.11 | 1.01 | 0.05 | 0.06 | 0.39 | 0.17 | 0.87 | 0.02 | 0.11 |
| Ash Wednesday | 2.56 | 1.55 | 4.21 | <0.005 | 0.00 | 1.68 | 0.94 | 2.99 | 0.08 | 0.05 | 1.14 | 0.63 | 2.07 | 0.67 | 0.10 |
| Easter | 2.56 | 1.35 | 4.83 | <0.005 | 0.00 | 2.35 | 1.07 | 5.16 | 0.03 | 0.06 | 1.65 | 0.83 | 3.27 | 0.15 | 0.10 |

| Positives | | | | | | | | | | | | | | | |
| --- | --- | --- | --- | --- | --- | --- | --- | --- | --- | --- | --- | --- | --- | --- | --- |
| All Ages | | | | | | | | | | | | | | | |
| Christmas | 0.25 | 0.11 | 0.59 | <0.005 | 0.00 | 0.08 | 0.01 | 0.56 | 0.01 | 0.15 | 0.64 | 0.21 | 1.96 | 0.44 | 0.35 |
| Ash Wednesday | 8.28 | 5.16 | 13.27 | <0.005 | 0.01 | 4.71 | 2.30 | 9.66 | <0.005 | 0.15 | 1.40 | 0.87 | 2.26 | 0.16 | 0.35 |
| Easter | 1.23 | 0.43 | 3.49 | 0.70 | 0.00 | 1.13 | 0.47 | 2.71 | 0.78 | 0.15 | 1.44 | 0.55 | 3.73 | 0.46 | 0.35 |
| 5-24 | | | | | | | | | | | | | | | |
| Christmas | 0.00 | 0.00 | 0.00 | <0.005 | 0.01 | 0.00 | 0.00 | 0.00 | <0.005 | 0.16 | 0.00 | 0.00 | 0.00 | 0.00 | 0.39 |
| Ash Wednesday | 10.27 | 6.10 | 17.30 | <0.005 | 0.01 | 5.82 | 2.84 | 11.92 | <0.005 | 0.16 | 1.46 | 0.93 | 2.30 | 0.10 | 0.39 |
| Easter | 0.63 | 0.17 | 2.36 | 0.49 | 0.00 | 0.74 | 0.19 | 2.83 | 0.66 | 0.15 | 1.14 | 0.18 | 7.22 | 0.89 | 0.39 |
| 25-44 | | | | | | | | | | | | | | | |
| Christmas | 1.50 | 0.63 | 3.59 | 0.36 | 0.00 | 0.59 | 0.15 | 2.34 | 0.45 | 0.15 | 2.24 | 0.69 | 7.23 | 0.18 | 0.28 |
| Ash Wednesday | 4.43 | 2.13 | 9.20 | <0.005 | 0.01 | 2.85 | 1.10 | 7.44 | 0.03 | 0.15 | 0.98 | 0.42 | 2.29 | 0.95 | 0.28 |
| Easter | 1.19 | 0.39 | 3.68 | 0.76 | 0.00 | 1.12 | 0.44 | 2.87 | 0.81 | 0.15 | 0.79 | 0.27 | 2.31 | 0.66 | 0.28 |

* Values shown in blue and red signify dampening (RR<1) and amplified (RR>1) effects.

Supplemental Table S11. Weekly counts and averages for four influenza health outcomes (tests, total positives, influenza A positives, and influenza B positives) and six age groups (all ages, ≤ 4, 5-24, 25-44, 45-64, and ≥ 65 years) for the Super Bowl (A), Ash Wednesday (B), and President's Day (C) (as compared to non-holiday weeks) in Milwaukee, WI (2004-2009).

| A | All Study Period | | | Weeks Without Sporting Events | | | Weeks of Super Bowl | | | MW Test | Percent Change* |
| --- | --- | --- | --- | --- | --- | --- | --- | --- | --- | --- | --- |
|  | (258 Weeks) | | | (217 Weeks) | | | (5 Weeks) | | |  |  |
|  | Counts | Mean | SD | Counts | Mean | SD | Counts | Mean | SD | P-Value | % |
| Tests | | | | | | | | | | | |
| All Ages | 2378 | 9.22 | 9.24 | 1678 | 11.99 | 10.18 | 125 | 25.00 | 10.82 | <0.005 | 166 |
| ≤ 4 | 527 | 2.04 | 2.07 | 316 | 2.26 | 2.16 | 5 | 1.00 | 1.73 | 0.25 | -51 |
| 5-24 | 1153 | 4.47 | 6.12 | 901 | 6.44 | 7.04 | 91 | 18.20 | 8.84 | <0.005 | 297 |
| 25-44 | 291 | 1.13 | 1.70 | 208 | 1.49 | 1.94 | 18 | 3.60 | 2.07 | <0.005 | 215 |
| 45-64 | 227 | 0.88 | 1.66 | 133 | 0.95 | 1.39 | 2 | 0.40 | 0.89 | 0.35 | -57 |
| ≥ 65 | 178 | 0.69 | 1.74 | 120 | 0.86 | 2.05 | 9 | 1.80 | 2.95 | 0.16 | 152 |
| Positive | | | | | | | | | | | |
| All Ages | 505 | 1.96 | 4.68 | 405 | 2.89 | 5.66 | 61 | 12.20 | 5.59 | <0.005 | 496 |
| ≤ 4 | 12 | 0.05 | 0.23 | 11 | 0.08 | 0.30 | 1 | 0.20 | 0.45 | 0.12 | 295 |
| 5-24 | 326 | 1.26 | 3.39 | 268 | 1.91 | 4.02 | 42 | 8.40 | 4.04 | <0.005 | 542 |
| 25-44 | 87 | 0.34 | 0.92 | 66 | 0.47 | 1.10 | 11 | 2.20 | 1.64 | <0.005 | 528 |
| 45-64 | 32 | 0.12 | 0.56 | 20 | 0.14 | 0.56 | 2 | 0.40 | 0.89 | 0.32 | 189 |
| ≥ 65 | 47 | 0.18 | 1.03 | 40 | 0.29 | 1.37 | 5 | 1.00 | 1.73 | 0.02 | 417 |
| Influenza A | | | | | | | | | | | |
| All Ages | 410 | 1.59 | 4.10 | 349 | 2.49 | 5.20 | 57 | 11.40 | 6.54 | <0.005 | 601 |
| ≤ 4 | 11 | 0.04 | 0.22 | 10 | 0.07 | 0.28 | 1 | 0.20 | 0.45 | 0.09 | 334 |
| 5-24 | 272 | 1.05 | 2.93 | 235 | 1.68 | 3.71 | 38 | 7.60 | 4.93 | <0.005 | 605 |
| 25-44 | 67 | 0.26 | 0.81 | 54 | 0.39 | 0.99 | 11 | 2.20 | 1.64 | <0.005 | 753 |
| 45-64 | 21 | 0.08 | 0.38 | 16 | 0.11 | 0.48 | 2 | 0.40 | 0.89 | 0.20 | 357 |
| ≥ 65 | 38 | 0.15 | 0.97 | 34 | 0.24 | 1.30 | 5 | 1.00 | 1.73 | <0.005 | 558 |
| Influenza B | | | | | | | | | | | |
| All Ages | 95 | 0.37 | 1.20 | 56 | 0.40 | 1.02 | 4 | 0.10 | 0.44 | 0.13 | -77 |
| ≤ 4 | 1 | 0.00 | 0.06 | 1 | 0.01 | 0.08 | 0 | 0.00 | 0.00 | 0.88 | -100 |
| 5-24 | 54 | 0.21 | 0.85 | 33 | 0.24 | 0.62 | 4 | 0.10 | 0.44 | 0.04 | -58 |
| 25-44 | 20 | 0.08 | 0.38 | 12 | 0.09 | 0.41 | 0 | 0.00 | 0.00 | 0.59 | -100 |
| 45-64 | 11 | 0.04 | 0.33 | 4 | 0.03 | 0.17 | 0 | 0.00 | 0.00 | 0.71 | -100 |
| ≥ 65 | 9 | 0.03 | 0.25 | 6 | 0.04 | 0.29 | 0 | 0.00 | 0.00 | 0.71 | -100 |

* Percent Change = ((Mean Cases Super Bowl Weeks – Mean Cases No Sporting Events Weeks) / Mean Cases No Sporting Events Weeks)*100%

| B | All Study Period | | | Weeks Without Christian Holidays | | | Weeks of Ash Wednesday | | | MW Test | Percent Change* |
| --- | --- | --- | --- | --- | --- | --- | --- | --- | --- | --- | --- |
|  | (258 Weeks) | | | (217 Weeks) | | | (5 Weeks) | | |  |  |
|  | Counts | Mean | SD | Counts | Mean | SD | Counts | Mean | SD | P-Value | % |
| Tests | | | | | | | | | | | |
| All Ages | 2378 | 9.22 | 9.24 | 2139 | 8.84 | 8.90 | 151 | 30.20 | 5.76 | <0.005 | 242 |
| ≤ 4 | 527 | 2.04 | 2.07 | 509 | 2.10 | 2.08 | 6 | 1.20 | 2.68 | 0.18 | -43 |
| 5-24 | 1153 | 4.47 | 6.12 | 1001 | 4.14 | 5.57 | 117 | 23.40 | 6.11 | <0.005 | 466 |
| 25-44 | 291 | 1.13 | 1.70 | 259 | 1.07 | 1.68 | 14 | 2.80 | 1.64 | 0.01 | 162 |
| 45-64 | 227 | 0.88 | 1.66 | 210 | 0.87 | 1.64 | 6 | 1.20 | 1.30 | 0.35 | 38 |
| ≥ 65 | 178 | 0.69 | 1.74 | 158 | 0.65 | 1.70 | 8 | 1.60 | 3.05 | 0.51 | 145 |
| Positive | | | | | | | | | | | |
| All Ages | 505 | 1.96 | 4.68 | 419 | 1.73 | 4.40 | 71 | 14.20 | 6.42 | <0.005 | 720 |
| ≤ 4 | 12 | 0.05 | 0.23 | 11 | 0.05 | 0.23 | 1 | 0.20 | 0.45 | 0.09 | 340 |
| 5-24 | 326 | 1.26 | 3.39 | 267 | 1.10 | 3.12 | 55 | 11.00 | 5.39 | <0.005 | 897 |
| 25-44 | 87 | 0.34 | 0.92 | 75 | 0.31 | 0.92 | 7 | 1.40 | 1.14 | <0.005 | 352 |
| 45-64 | 32 | 0.12 | 0.56 | 26 | 0.11 | 0.53 | 3 | 0.60 | 0.89 | <0.005 | 458 |
| ≥ 65 | 47 | 0.18 | 1.03 | 39 | 0.16 | 1.03 | 5 | 1.00 | 1.73 | 0.01 | 521 |
| Influenza A | | | | | | | | | | | |
| All Ages | 410 | 1.59 | 4.10 | 338 | 1.40 | 3.80 | 66 | 13.20 | 5.89 | <0.005 | 845 |
| ≤ 4 | 11 | 0.04 | 0.22 | 10 | 0.04 | 0.22 | 1 | 0.20 | 0.45 | 0.07 | 384 |
| 5-24 | 272 | 1.05 | 2.93 | 220 | 0.91 | 2.65 | 51 | 10.20 | 4.66 | <0.005 | 1022 |
| 25-44 | 67 | 0.26 | 0.81 | 55 | 0.23 | 0.80 | 7 | 1.40 | 1.14 | <0.005 | 516 |
| 45-64 | 21 | 0.08 | 0.38 | 19 | 0.08 | 0.37 | 2 | 0.40 | 0.89 | 0.16 | 409 |
| ≥ 65 | 38 | 0.15 | 0.97 | 33 | 0.14 | 0.97 | 5 | 1.00 | 1.73 | <0.005 | 633 |
| Influenza B | | | | | | | | | | | |
| All Ages | 95 | 0.37 | 1.20 | 81 | 0.33 | 1.15 | 5 | 1.00 | 1.73 | 0.07 | 199 |
| ≤ 4 | 1 | 0.00 | 0.06 | 1 | 0.00 | 0.06 | 0 | 0.00 | 0.00 | 0.89 | -100 |
| 5-24 | 54 | 0.21 | 0.85 | 47 | 0.19 | 0.83 | 4 | 0.80 | 1.30 | 0.02 | 312 |
| 25-44 | 20 | 0.08 | 0.38 | 20 | 0.08 | 0.39 | 0 | 0.00 | 0.00 | 0.61 | -100 |
| 45-64 | 11 | 0.04 | 0.33 | 7 | 0.03 | 0.28 | 1 | 0.20 | 0.45 | <0.005 | 591 |
| ≥ 65 | 9 | 0.03 | 0.25 | 6 | 0.02 | 0.22 | 0 | 0.00 | 0.00 | 0.77 | -100 |

* Percent Change = ((Mean Cases Ash Wednesday Weeks – Mean Cases No Christian Holidays Weeks) / Mean Cases No Christian Holidays Weeks)*100%

| C | All Study Period | | | Weeks Without Federal Holidays | | | Weeks of President’s Day | | | MW Test | Percent Change* |
| --- | --- | --- | --- | --- | --- | --- | --- | --- | --- | --- | --- |
|  | (258 Weeks) | | | (213 Weeks) | | | (5 Weeks) | | |  |  |
|  | Counts | Mean | SD | Counts | Mean | SD | Counts | Mean | SD | P-Value | % |
| Tests | | | | | | | | | | | |
| All Ages | 2378 | 9.22 | 9.24 | 2020 | 9.48 | 9.49 | 128 | 25.60 | 4.98 | <0.005 | 170 |
| ≤ 4 | 527 | 2.04 | 2.07 | 458 | 2.15 | 2.07 | 11 | 2.20 | 3.35 | 0.68 | 2 |
| 5-24 | 1153 | 4.47 | 6.12 | 967 | 4.54 | 6.13 | 99 | 19.80 | 4.02 | <0.005 | 336 |
| 25-44 | 291 | 1.13 | 1.70 | 249 | 1.17 | 1.80 | 12 | 2.40 | 0.89 | 0.01 | 105 |
| 45-64 | 227 | 0.88 | 1.66 | 193 | 0.91 | 1.76 | 3 | 0.60 | 0.55 | 0.88 | -34 |
| ≥ 65 | 178 | 0.69 | 1.74 | 151 | 0.71 | 1.83 | 3 | 0.60 | 0.89 | 0.68 | -15 |
| Positive | | | | | | | | | | | |
| All Ages | 505 | 1.96 | 4.68 | 429 | 2.01 | 4.83 | 54 | 10.80 | 5.36 | <0.005 | 436 |
| ≤ 4 | 12 | 0.05 | 0.23 | 10 | 0.05 | 0.23 | 0 | 0.00 | 0.00 | 0.64 | -100 |
| 5-24 | 326 | 1.26 | 3.39 | 275 | 1.29 | 3.45 | 43 | 8.60 | 5.32 | <0.005 | 566 |
| 25-44 | 87 | 0.34 | 0.92 | 76 | 0.36 | 0.97 | 7 | 1.40 | 1.34 | 0.01 | 292 |
| 45-64 | 32 | 0.12 | 0.56 | 28 | 0.13 | 0.60 | 2 | 0.40 | 0.55 | 0.01 | 204 |
| ≥ 65 | 47 | 0.18 | 1.03 | 39 | 0.18 | 1.12 | 2 | 0.40 | 0.55 | 0.01 | 118 |
| Influenza A | | | | | | | | | | | |
| All Ages | 410 | 1.59 | 4.10 | 342 | 1.61 | 4.23 | 47 | 9.40 | 4.39 | <0.005 | 485 |
| ≤ 4 | 11 | 0.04 | 0.22 | 9 | 0.04 | 0.22 | 0 | 0.00 | 0.00 | 0.66 | -100 |
| 5-24 | 272 | 1.05 | 2.93 | 225 | 1.06 | 2.96 | 39 | 7.80 | 4.60 | <0.005 | 638 |
| 25-44 | 67 | 0.26 | 0.81 | 59 | 0.28 | 0.84 | 5 | 1.00 | 1.41 | 0.09 | 261 |
| 45-64 | 21 | 0.08 | 0.38 | 18 | 0.08 | 0.40 | 1 | 0.20 | 0.45 | 0.19 | 137 |
| ≥ 65 | 38 | 0.15 | 0.97 | 30 | 0.14 | 1.05 | 2 | 0.40 | 0.55 | <0.005 | 184 |
| Influenza B | | | | | | | | | | | |
| All Ages | 95 | 0.37 | 1.20 | 87 | 0.41 | 1.28 | 7 | 1.40 | 1.67 | 0.01 | 243 |
| ≤ 4 | 1 | 0.00 | 0.06 | 1 | 0.00 | 0.07 | 0 | 0.00 | 0.00 | 0.88 | -100 |
| 5-24 | 54 | 0.21 | 0.85 | 50 | 0.23 | 0.92 | 4 | 0.80 | 0.84 | <0.005 | 241 |
| 25-44 | 20 | 0.08 | 0.38 | 17 | 0.08 | 0.39 | 2 | 0.40 | 0.89 | 0.12 | 401 |
| 45-64 | 11 | 0.04 | 0.33 | 10 | 0.05 | 0.36 | 1 | 0.20 | 0.45 | 0.02 | 326 |
| ≥ 65 | 9 | 0.03 | 0.25 | 9 | 0.04 | 0.28 | 0 | 0.00 | 0.00 | 0.70 | -100 |

* Percent Change = ((Mean Cases President’s Day Weeks – Mean Cases No Federal Holidays Weeks) / Mean Cases No Federal Holidays Weeks)*100%
